# Specification of human germ cell fate with enhanced progression capability supported by hindgut organoids

**DOI:** 10.1016/j.celrep.2022.111907

**Published:** 2023-01-05

**Authors:** João Pedro Alves-Lopes, Frederick CK Wong, Walfred WC Tang, Wolfram H Gruhn, Navin B Ramakrishna, Geraldine M Jowett, Kirsi Jahnukainen, M Azim Surani

**Affiliations:** 1 Wellcome/Cancer Research UK Gurdon Institute, University of Cambridge, Tennis Court Road, Cambridge CB2 1QN, UK; 2 Department of Physiology, Development and Neuroscience, University of Cambridge, Downing Street, Cambridge CB2 3DY, UK; 3 NORDFERTIL Research Lab Stockholm, Childhood Cancer Research Unit, J9:30, Department of Women’s and Children’s Health, Karolinska Institutet and Karolinska University Hospital, Visionsgatan 4, Solna 17164, Stockholm, Sweden; 4 Genome Institute of Singapore, A*STAR, Biopolis, Singapore, 138672, Singapore; 5 New Children’s Hospital, Paediatric Research Centre, University of Helsinki and Helsinki University Hospital, Pl 281, 00029 Helsinki, Finland

## Abstract

Human primordial germ cells (hPGCs), the precursors of sperm and eggs, are specified during weeks 2−3 after fertilization. Few studies on ex vivo and in vitro cultured human embryos reported plausible hPGCs on embryonic day (E) 12−13, and in an E16−17 gastrulating embryo. In vitro, hPGC-like cells (hPGCLCs) can be specified from the intermediary pluripotent stage or peri-gastrulation precursors. Here, we explore the broad spectrum of hPGCLC precursors and how different precursors impact hPGCLC development. We show that resetting precursors can give rise to hPGCLCs (rhPGCLCs) in response to BMP. Strikingly, rhPGCLCs co-cultured with human hindgut organoids progress at a pace reminiscent of in vivo hPGC development, unlike those derived from peri-gastrulation precursors. Moreover, rhPGCLC specification depends on both EOMES and TBXT, not just on EOMES as for peri-gastrulation hPGCLCs. Importantly, our study provides the foundation for developing efficient *in vitro* models of human gametogenesis.

## Introduction

Primordial germ cells (PGCs), the precursors of gametes, transmit genetic and epigenetic (non-genetic modifications that influence gene expression) information to subsequent generations. Mouse PGC (mPGC) specification occurs during early post-implantation development, when epiblast cells exiting the naïve pluripotent state start to acquire PGC competence at ~E5.5^1^, before primitive streak formation. The state of competence continues until ~E6.75, coinciding with the appearance of the primitive streak^1^. Specification of mPGCs is triggered by BMP and WNT ligands from the surrounding extra-embryonic tissues at E6.25^1–3^. In vitro, a two-step model was established to recapitulate mPGC specification, using the in vivo developmental trajectory as guidance^4^. The mPGC-competent state transiently develops as epiblast-like cells (mEpiLCs) during the transition from naïve to primed pluripotency. Subsequently, BMP stimulation of mEpiLCs efficiently induces mPGC-like cells (mPGCLCs)^4^. In this specification context, the expression of T is a prerequisite for the activation of PGC transcription factors such as PRDM1^2,5^. Mouse PGCLCs showed high similarities to in vivo early migratory mPGCs and could undergo gametogenesis upon transplantation into mouse neonatal testis^4^ or adult ovaries^6^.

Specification of human PGCs (hPGCs) occurs at approximately between weeks 2−3 after fertilization. Studies on embryonic day (E) 12−13 human embryos (Carnegie Stage 5−6a: CS5−6a) identified few hPGCs^7^, which was recently supported by the detection of individual cells expressing key PGC-marker genes in cultured human embryos^8^. Moreover, a single-cell transcriptomic study on a rare E16−17 human embryo (CS7) reported a small number of hPGCs in the posterior region of the gastrulating epiblast^9^. At approximately E24, hPGCs were observed clustering in the extra-embryonic yolk sac wall near the allantois^10^ before migration commenced through the hindgut and dorsal mesentery into the developing gonads during weeks 4−6 (~E24−42). In the course of migration and gonadal colonization, hPGCs undergo an extensive epigenetic reprogramming, comprising global DNA demethylation and chromatin reorganization events^11,12^. Upon colonization of the primitive gonads, during weeks 7–10, hPGCs start to differentiate into pro-spermatogonia or oogonia in the developing testis or ovary, respectively^13^.

In vitro, human pluripotent stem cells (hPSCs) can undergo specification into hPGC-like cells (hPGCLCs) upon exposure to BMP^14^. Moreover, transient incipient mesoderm-like cells (iMeLCs)^14^ and precursors of mesendoderm (PreME)^15^, generated by differentiation of primed hPSCs towards mesendodermal fate, respond to BMP and can specify hPGCLCs. Both PreME and iMeLC precursors are considered to represent peri-gastrulation cell identities in vivo^13,16^. Similarly, self-renewing hPSCs cultured under the four-inhibitor (4i) condition can efficiently induce hPGCLCs. These 4i-hPSC precursors express early gastrulation-associated genes compared to their primed hPSC counterparts, and are therefore considered peri-gastrulation precursors^13,16,17^. On the other hand, two recent studies reported independent protocols to maintain hPSCs in intermediary states of pluripotency (between primed and naïve pluripotency)^18,19^. In one of these reports, the authors defined human chimera PSCs (hXPSCs) which could respond to BMP and specify hPGCLCs^19^, while in the other study, human formative stem cells (hFSCs) were not tested for this cell fate^18^.

Previous studies established culture conditions for naïve hPSCs either by direct derivation from human embryonic inner cell mass or by resetting primed hPSCs into naïve hPSCs^20–23^. The resetting process gradually establishes the transcriptomic and epigenetic profile of naïve pluripotency resembling the human pre-implantation epiblast ^20,22–25^. The extended nature of the resetting process enables hPSCs to acquire transient transcriptional profiles in-between primed and naïve pluripotency^25^, hereafter termed the “resetting” states. Similar states of transitional pluripotency are also expected during capacitation (conversion from naïve to primed pluripotency), hereafter termed the “capacitating” states ^26,27^.

In this study, we used both capacitating and resetting precursors to show that human embryonic stem cells (hESCs) transitioning between naïve to primed pluripotency (and vice versa) respond to BMP to generate hPGCLCs. Furthermore, we establish a human hindgut co-culture system promoting hPGCLC development to show that hPGCLCs derived from resetting precursors harbour an enhanced progression capability compared to their peri-gastrulation counterparts, which occurs at a tempo reminiscent of the timings observed in vivo. Remarkably, the transcription factors EOMES and TBXT are both required to specify hPGCLCs from resetting precursors, whereas only EOMES is essential for specification from peri-gastrulation precursors. This suggests that distinct gene regulatory networks drive the onset of PGC specification from pre-and peri-gastrulation precursors. Our study explores in vitro approaches to specify and progress hPGCLCs with enhanced progression capabilities, creating opportunities to investigate the mechanisms of hPGC progression, the window for hPGC specification, and the determinants of cell-fate decisions during early human development.

## Results

### Specification of hPGCLCs from capacitating and resetting precursors

**Figure 1:**
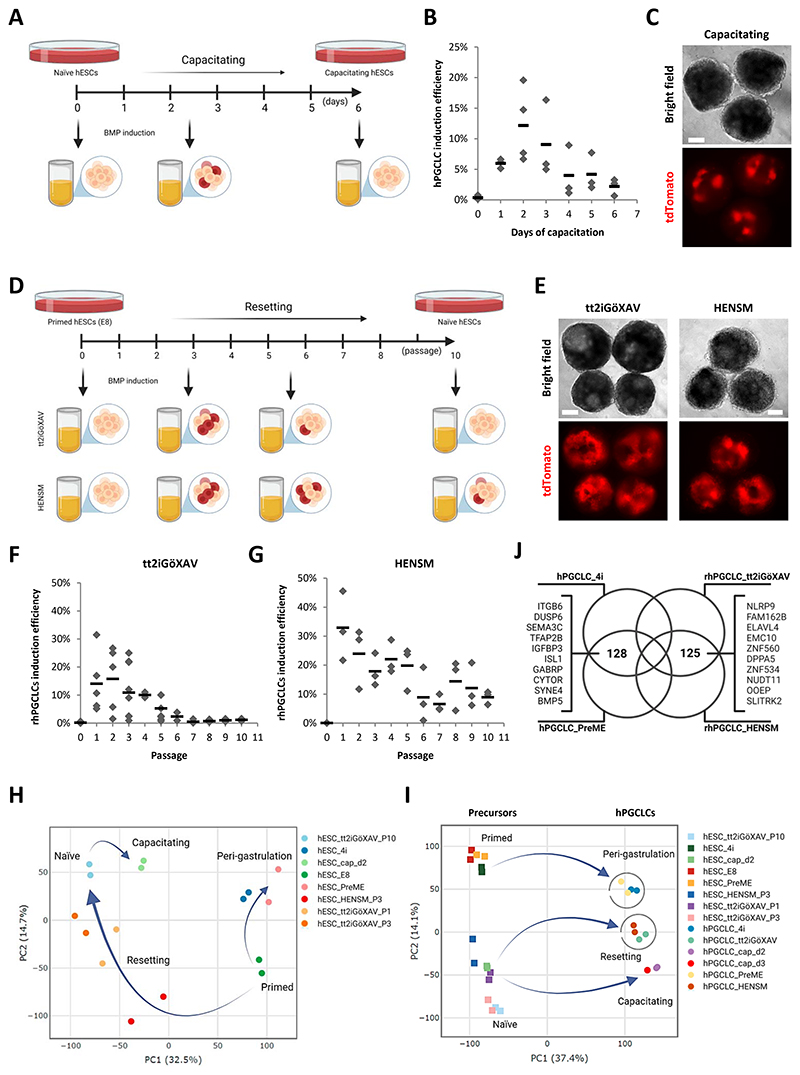
hPGCLC specification from capacitating and resetting precursors (A) Schematic diagram for capacitation of naïve hESCs; capacitating hESCs are competent for hPGCLC fate. (B) Efficiency of hPGCLC induction (% of hPGCLCs in day 4 embryoid bodies) from capacitating hESCs over six days of capacitation protocol, measured by the co-expression of NANOS3−tdTomato and TNAP on flow cytometry analysis. Horizontal bars represent the mean percentage for each day. At least n=3 measurements were taken from independent experiments for each time point. (C) Day 4 embryoid bodies generated from capacitating NANOS3−tdTomato hESCs. Scale bar: 200 um. (D) Schematic diagram for resetting (tt2iGöXAV and HENSM) primed hESCs into naïve hESCs; resetting hESCs are competent for hPGCLC specification. (E) Day 4 embryoid bodies from resetting (tt2iGöXAV and HENSM) NANOS3−tdTomato hESCs. Scale bar, 200 um. (F and G) Efficiency of hPGCLC induction (% of hPGCLCs in day 4 embryoid bodies) from resetting tt2iGöXAV (F) and HENSM (G) hESCs over 10 passages, measured by the co-expression of NANOS3−tdTomato and TNAP on flow cytometry analysis. P0 represents the induction efficiency of primed hESCs (cultured in E8 medium). Horizontal bars represent the mean percentage for each day. At least n=3 measurements were taken from independent experiments for each time point. (H) Two-dimensional principal component analysis for hESCs and hPGCLC precursors from peri-gastrulation (4i and PreME), resetting tt2iGöXAV, resetting HENSM, and capacitating (cap) conditions. Passage after conversions (P) or days of capacitation (d) are indicated. Arrows show potential conversion trajectories. (I) Two-dimensional principal component analysis for hESCs, hPGCLC precursors, and day 4 hPGCLCs from peri-gastrulation (4i and PreME), resetting tt2iGöXAV, resetting HENSM, and capacitating (cap) conditions. Passage after conversions (P) or days of capacitation (d) are indicated. Arrows show potential specification trajectories. (J) Venn diagram showing differentially expressed genes (log_2_FC > 2 and adjusted p-value < 0.05) commonly upregulated in day 4 peri-gastrulation hPGCLCs (4i and PreME) versus rhPGCLCs (tt2iGöXAV and HENSM), and vice versa. See also Figures S1, S2, S3, S4, and S5.

To investigate hPGCLC competence in hESCs transitioning from naïve to primed pluripotency, we first reset a primed NANOS3−tdTomato hESC reporter line (W15)^15^ to the naïve pluripotent state using a previously described protocol (tt2iGöXAV)^22^. Fully reset naïve hESCs, requiring approximately 10 passages (P) after conversion in tt2iGöXAV conditions, exhibited very low potential for the hPGCLC fate (~1%, Figure 1A and 1B). Subsequently, we allowed these fully reset hESCs (P10) to exit their naïve state by capacitating these cells towards the primed state of pluripotency ^26^ and tested their response to BMP during the capacitation process (Figure 1A and Figure S1A). We observed a progressive increase in competence for hPGCLC specification between days one and two (~12%), which gradually declined to ~3% by the sixth day of capacitation (Figure 1B and 1C). We confirmed that these hPGCLCs showed expression of key hPGC markers, including SOX17, BLIMP1 (PRDM1), OCT4 (POU5F1), and NANOG (Figure S1D). Moreover, the hPGCLCs could be identified and separated from somatic cell populations in the embryoid bodies based on NANOS3−tdTomato (Figure 1C) and Tissue Non-specific Alkaline Phosphatase (TNAP) co-expression (Figure S1E), meeting previously described criteria^15^ for hPGCLCs specified from peri-gastrulation precursors (Figure S2A−S2H).

Prolonged culture in naïve conditions can result in genetic and epigenetic abnormalities^22,28,29^. To mitigate this possibility, we asked if there is a window of competence for the hPGCLC fate during the conversion of hESCs from the primed to the naïve state of pluripotency^25^. Here, we used two different resetting protocols (tt2iGöXAV^22^ and HENSM^23^) to convert primed NANOS3−tdTomato hESCs towards the naïve state (Figure S1B and S1C) and tested resetting hESCs during consecutive time points during the transition for their response to BMP (Figure 1D). We found that hESCs undergoing resetting conversion could specify into hPGCLCs, hereafter referred to as resetting hESC-derived hPGCLCs (rhPGCLCs) (Figure 1E−1G). For both resetting protocols, we found that hESCs gained a high competence for hPGCLC fate at P1. This was followed by a decrease in competence for rhPGCLC specification through to P10, which was more pronounced in tt2iGöXAV resetting hESCs (Figure 1F and 1G). Notably, both resetting precursors (tt2iGöXAV and HENSM) yielded rhPGCLCs expressing hPGC markers SOX17, PRDM1 (BLIMP1), POU5F1 (OCT4), and NANOG (Figure S1F and S1H) and could be isolated based on NANOS3−tdTomato/TNAP co-expression (Figure S1G and S1I), in the same way as described previously for hPGCLCs specified from peri-gastrulation precursors (Figure S2A−S2H). Moreover, we specified rhPGCLCs from both tt2iGöXAV and HENSM resetting precursors using our NANOS3-mCherry hESC reporter line (W24), which was previously used in our lab to obtain peri-gastrulation hPGCLCs^15,17^. These rhPGCLCs also expressed the hPGC markers SOX17, BLIMP1, OCT4, and NANOG (Figure S2I and S2J) and could be isolated based on Podoplanin (PDPN)^30^ and TNAP co-expression (Figure S2K and S2L).

To determine their relative position in the spectrum of human pluripotency^16,31^, we used principle component analysis (PCA) to compare the transcriptomic signatures of these newly-identified capacitating and resetting precursors with those from primed (E8), naïve (tt2iGöXAV, P10), and peri-gastrulation precursors (4i and PreME). Our clustering analysis revealed that while capacitating and resetting precursors occupy positions between primed and naïve pluripotency, the peri-gastrulation precursors cluster closer to the primed state (Figure 1H and 1I and Figure S3A). As expected, the separation of both capacitating and resetting precursors from peri-gastrulation precursors on PCA plots had the contribution from genes related to naïve pluripotency (e.g. *DPPA3, DNMT3L*, and *TBX3*) and peri-gastrulation state (e.g. *DUSP6, EOMES, GABRB2*, and *TBXT*), respectively (Figure S3B and Figure S4A).

To comprehensively profile the identity of hPGCLCs specified from capacitating and resetting precursors, we sequenced their transcriptomes and integrated these signatures with those of hPGCLCs obtained from peri-gastrulation precursors. All hPGCLCs analysed shared similar transcriptomic profiles, which were highly distinct from their hESC precursors (principle component 1 (PC1) in Figure 1I and Figure S3A). Specifically, differential expression of genes exclusively related to pluripotency (e.g. *SOX2* and *ZIC2*) and hPGC fate (e.g. *NANOS3* and *SOX17*) contributed to this segregation on PC1 (Figure S3C and and Figure S4A). However, we noticed that hPGCLCs clustered according to their precursors along PC2, which reflects ~14% of the explained variances in our system of samples (Figure 1I). The distribution pattern of hPGCLCs on PC2 is correlated with the corresponding precursors, suggesting partial maintenance of precursor-specific transcriptomic signatures in the resulting hPGCLCs (Figure 1I and Figure S3C). We then focused our analysis on the differences between rhPGCLCs (tt2iGöXAV and HENSM) and peri-gastrulation hPGCLCs (4i and PreME) and identified a set of 125 differentially expressed genes upregulated in rhPGCLCs (Figure 1J); among these, some were also detected in the hPGCs form CS7 human embryo data set (e.g *NLRP9, FAM162B, DPPA5*, and *ELAVL4*; Figure S4A). On the other hand, we identified 128 differentially expressed genes upregulated in peri-gastrulation hPGCLCs (Figure 1J), from which some are associated with the amniotic^9,24^ (e.g. *GABRP, ITGB6, ISL1*, and *SEMA3C*) and mesodermal^9^ lineages (e.g. *CYTOR, BMP5* and *IGFBP3*; Figure S4A), respectively.

A few days after their specification, mouse^32^, pig^15^, and cynomolgus monkey^30^ PGCs initiate an extensive epigenetic reprogramming, including a progressive loss of 5mC and H3K9me2 markers to impressively low levels. To understand if the epigenetic reprogramming had been initiated in our newly specified rhPGCLCs, we stained day 4 embryoid bodies for 5mC and H3K9me2 (Figure S5A−5D). We found that compared with their neighbouring somatic cells, hPGCLCs derived from either resetting or peri-gastrulation precursors had lower levels of 5mC and H3K9me2. However, the reduction of these two epigenetic markers is generally more pronounced in rhPGCLCs, suggesting a more advanced state of their epigenetic reprogramming (Figure S5A−5D).

### Specification of resetting hPGCLCs is dependent on BMP and SOX17

**Figure 2:**
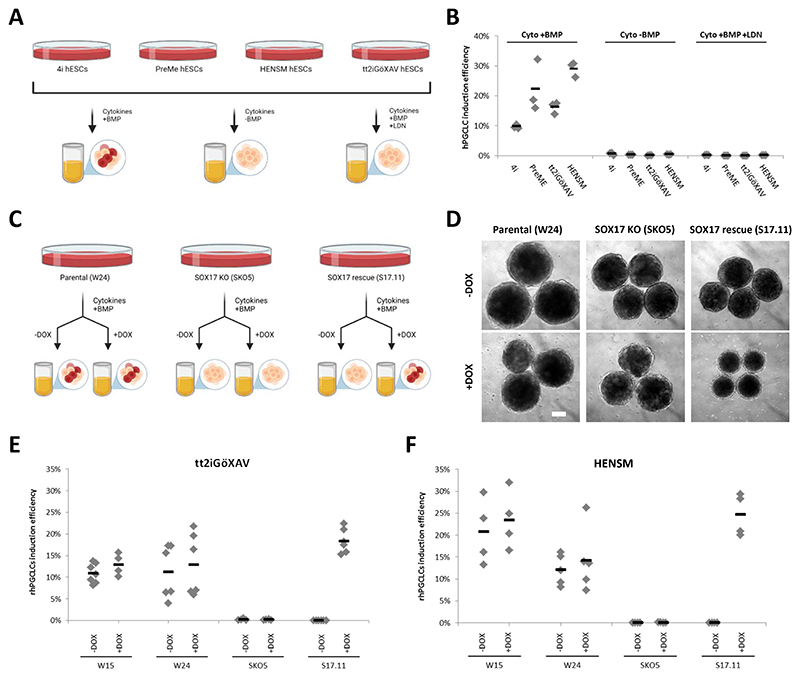
Dependency of BMP and SOX17 for hPGCLC specification from resetting precursors (A) Schematic diagram for the BMP dependency experiment. (B) Efficiency of hPGCLC induction (% of hPGCLCs in day 4 embryoid bodies) from peri-gastrulation (4i and PreME) and resetting (tt2iGöXAV and HENSM) hESCs under three experimental conditions (Cyto+BMP, Cyto-BMP, and Cyto+BMP+LDN), measured by the co-expression of NANOS3−tdTomato and TNAP on flow cytometry analysis. Horizontal bars represent the mean percentage for each condition. At least n=3 measurements were taken from independent experiments for each condition. (C) Schematic diagram for the SOX17 dependency experiment. (D) Bright-field images for day 4 embryoid bodies generated from HENSM resetting hESCs with three different genetic backgrounds (parental (W24), SOX17 knockout (SKO5), and SOX17 rescue (S17.11) lines), under the absence or presence of DOX. Scale bar, 200 um. (E and F) Efficiency of hPGCLC induction (% of hPGCLCs in day 4 embryoid bodies) from (E) tt2iGöXAV and (F) HENSM resetting hESCS in four different genetic backgrounds (W15, parental W24, SOX17 knockout (SKO5), and SOX17 rescue (S17.11) lines), under the absence or presence of DOX, measured by the co-expression of PDPN and TNAP on flow cytometry analysis. Horizontal bars represent the mean percentage for each condition. At least n=4 measurements were taken from independent experiments for each condition. See also Figures S6 and S7.

Previously described hPGCLCs specified from peri-gastrulation precursors depend on BMP as an inducing factor^14,15,33^, and SOX17 as the master transcription factor to generate the hPGCLC identity ^15,17,34^. To investigate if rhPGCLCs specification requires BMP, we induced hPGCLC fate from resetting (tt2iGöXAV and HENSM) and peri-gastrulation (4i and PreME) precursors: (i) in the presence of BMP, (ii) in the presence of BMP and a BMP inhibitor (LDN), or (iii) in the absence of BMP to rule out the possibility that endogenous BMP could trigger hPGCLC specification (Figure 2A). Identical to peri-gastrulation hPGCLCs, rhPGCLCs required exogenous BMP to initiate specification (Figure 2B, Figure S6A and S6B).

To determine if rhPGCLCs specification requires SOX17, we first induced hPGCLC fate from resetting (tt2iGöXAV and HENSM) precursors in a SOX17 knockout hESC line (SKO5), previously generated in our lab^17^ (Figure 2C). After four days of induction, no hPGCLCs were detected in healthy EBs generated from both tt2iGöXAV and HENSM resetting precursors (Figure 2D−2F, Figure S7A). Next, we introduced a doxycycline (DOX)-inducible *SOX17* transgene in the SKO5 background to generate a rescue line (S17.11). Under the presence of DOX, we could rescue rhPGCLC specification when S17.11 was induced to rhPGCLC fate from both tt2iGöXAV and HENSM precursors (Figure 2D−2F, Figure S7A), demonstrating that hPGCLC specification from resetting precursors also require SOX17 expression.

### Resetting hPGCLCs have greater progression capability than peri-gastrulation hPGCLCs

#### Human hindgut organoids

**Figure 3:**
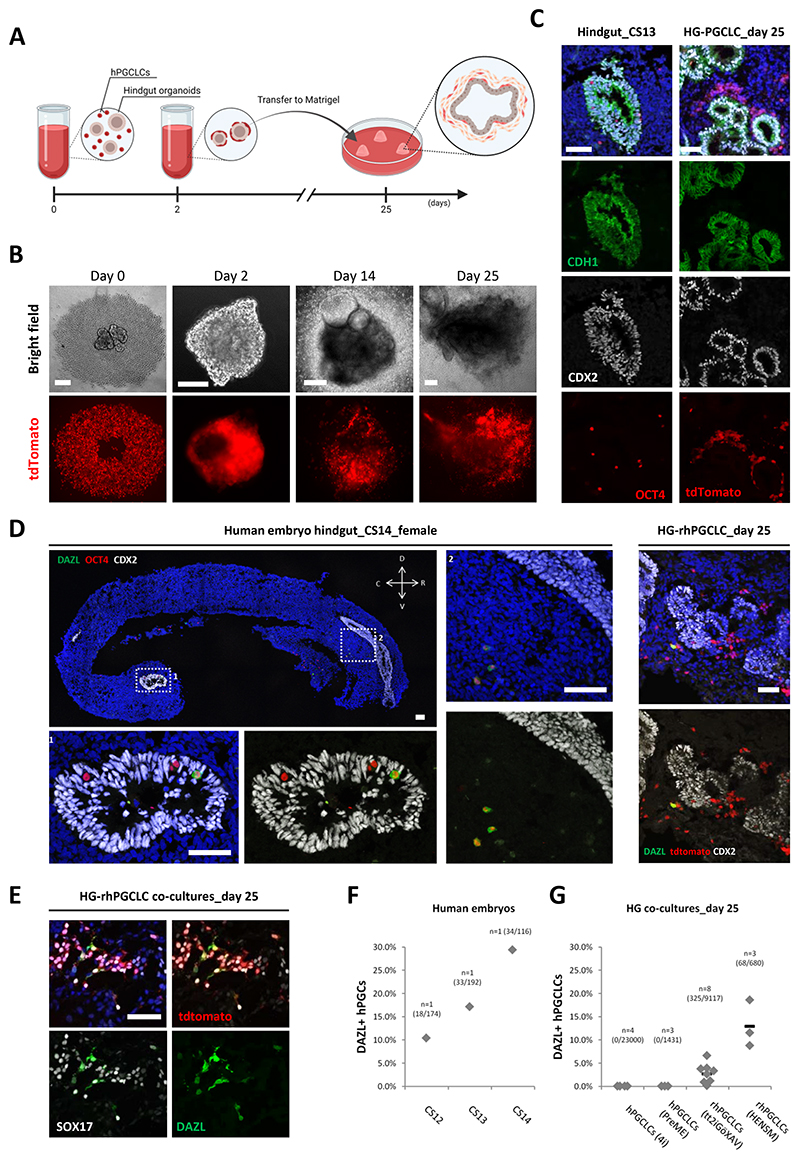
Progression of resetting hPGCLCs supported by human hindgut organoid co-cultures (A) Schematic diagram for the co-culture strategy of hPGCLCs with human hindgut organoids. (B) Co-culture of human hindgut organoid and NANOS3−tdTomato resetting (tt2iGöXAV) hPGCLCs over 25 days. Scale bar, 200 um (except day 2: 100 um). (C) Immunofluorescence of OCT4, CDH1, and CDX2 on sections of a CS13 human hindgut (left panel) and Immunofluorescence of CDH1 and CDX2 on section of a day 25 human hindgut organoid (HG) co-culture containing resetting (tt2iGöXAV) hPGCLCs expressing NANOS3−tdTomato (right panel). DAPI nuclear counterstain showed in blue. Scale bar, 50 um. (D) Immunofluorescence of OCT4, CDX2, and DAZL on sections of a CS14 human hindgut (left panel) and Immunofluorescence of CDX2 and DAZL on section of a day 25 human hindgut organoid (HG) co-culture containing resetting (tt2iGöXAV) hPGCLCs expressing NANOS3−tdTomato (right panel). DAPI nuclear counterstain showed in blue. Scale bar, 50 um. (E) Immunofluorescence of SOX17 and DAZL on section of a day 25 human hindgut organoid (HG) co-culture containing resetting (tt2iGöXAV) hPGCLCs expressing NANOS3−tdTomato. DAPI nuclear counterstain showed in blue. Scale bar, 50 um. (F) Percentage of DAZL positive hPGCs out of Oct4 positive hPGCs in CS12−CS14 human embryos. Total number of cells counted (double DAZL and OCT4 positive/OCT4 positive cells) per condition shown between brackets. Only one human embryo for each stage was analysed due to the rarity of these samples. (G) Percentage of DAZL positive peri-gastrulation (4i or PreME) or resetting (tt2iGöXAV or HENSM) hPGCLCs out of NANOS3-tdTomato positive hPGCLCs in day 25 human hindgut organoid (HG) co-cultures. Horizontal bars represent the mean percentage for each condition. At least n=3 measurements were taken from independent experiments for each condition. Total number of cells counted (double DAZL and tdTomato positive/tdTomato positive cells) per condition shown between brackets. See also Figures S8, S9, S10 and S11.

After specification, hPGCs migrate through the hindgut and the dorsal mesentery before colonizing the genital ridges^10,35^. While the specification of rhPGCLCs captures some early stages of hPGC progression, we hypothesised that co-cultures with human hindgut organoids would provide an elegant and xeno-free approach to promote and physiologically recapitulate further hPGCLC development in vitro (Figure 3A).

Hindgut spheroids were derived from hESCs by first inducing their differentiation into definitive endoderm^15^ (Figure S8A and S8B) and then into posterior endoderm hindgut (HG) expressing CDX2 (Figure S8A, S8C, and S8D)^36,37^. The HG spheroids were aggregated with either rhPGCLCs (tt2iGöXAV or HENSM) or peri-gastrulation hPGCLCs (4i or PreME) in ultra-low attachment 96-well plates for 2 days, and then transferred into Matrigel for an additional 23 days of culture (Figure 3A and 3B and Figure S8E). The hPGCLCs started aggregating tightly on the surface of the HGs; from approximately day 7 onwards, mesenchymal-like cells originated from the HGs^36,37^ started to invade Matrigel followed by hPGCLCs, which became widely distributed throughout the co-cultures (Figure 3B and Figure S8E). At the end of the culture period, hPGCLCs were mainly localized in the surrounding mesenchymal tissue and on the surface of the HG epithelium (Figure 3C), which expressed the specific hindgut markers CDX2 and CDH1 (Figure S9A)^36,37^. Less frequently, we also found hPGCLCs in the HG epithelium (Figure S9B), mirroring some migratory hPGCs observed in vivo (Figure 3C).

Notably, we detected differences in the distribution pattern and morphology between resetting and peri-gastrulation hPGCLCs. While rhPGCLCs dispersed as individually recognisable cells with cytoplasmic protrusions characteristic of migratory hPGCs^35^, peri-gastrulation hPGCLCs remained as tight clumps with indistinguishable individual cells (Figure S8F). To further investigate their distribution pattern, we co-cultured either rhPGCLCs or peri-gastrulation hPGCLCs with HGs in ultra-low attachment wells, in the absence of Matrigel, for 11 days to explore direct hPGCLC−HG interaction in the absence of exogenously-supplied extracellular matrix. We observed that rhPGCLCs started to invade and colonize the HG surface as early as day 3, which became more evident during days 5−11. On the other hand, peri-gastrulation hPGCLCs remained as tight cellular aggregates for the entire culture period, suggesting a reduced interaction and integration with HGs (Figure S8G).

During migration, hPGCs proceed with resetting their epigenome^11,12^ while asynchronously upregulating transcripts for progression markers, including DAZL^38^. Here, we examined the migratory hPGCs in CS12−CS14 *ex vivo* human embryos and confirmed the presence of DAZL protein in some of these cells (Figure S9C and Figure S10A−D). Specifically, we observed an increasing percentage of DAZL-positive hPGCs from CS12 to CS14 (10−29%) migrating through the hindgut (CDX2 positive epithelium) and dorsal mesentery (Figure 3D and 3F, and Figure S10A−D). Importantly, 3−13% of rhPGCLCs (tt2iGöXAV or HENSM) showed expression of DAZL when co-cultured with HGs (Figure 3D and 3E, and Figure S11A and S11B). By contrast, peri-gastrulation hPGCLCs (4i or PreME) did not show DAZL expression under the same co-culture conditions (Figure 3G and Figure S11A). Notably, DAZL up-regulation was progressive; there were no rhPGCLCs expressing DAZL during the first 14 days of culture, before an increase in expression by day 25 (Figure S11C). Additionally, we found DDX4 expression in DAZL-positive rhPGCLCs co-cultured with HG (Figure S11D), which is consistent with DDX4 expression detected in human^38^, rhesus macaque^39^, and cynomolgus monkey^30^ migratory PGCs. Another critical event in hPGC development is the epigenetic reprogramming occurring during migration and early gonadal colonization^11,12^. DAZL-positive rhPGCLCs present in the HG co-cultures exhibited low 5mC and H3K9me2 (Figure S11E and S11F), pointing towards a progressive epigenetic reprogramming, as observed in vivo in human^11^, rhesus macaque^39^, and cynomolgus monkey^30^ germline. Together, these results support the notion that DAZL upregulation in rhPGCLCs co-cultured with HGs is part of a coordinated programme for rhPGCLC progression in vitro.

**Figure 4:**
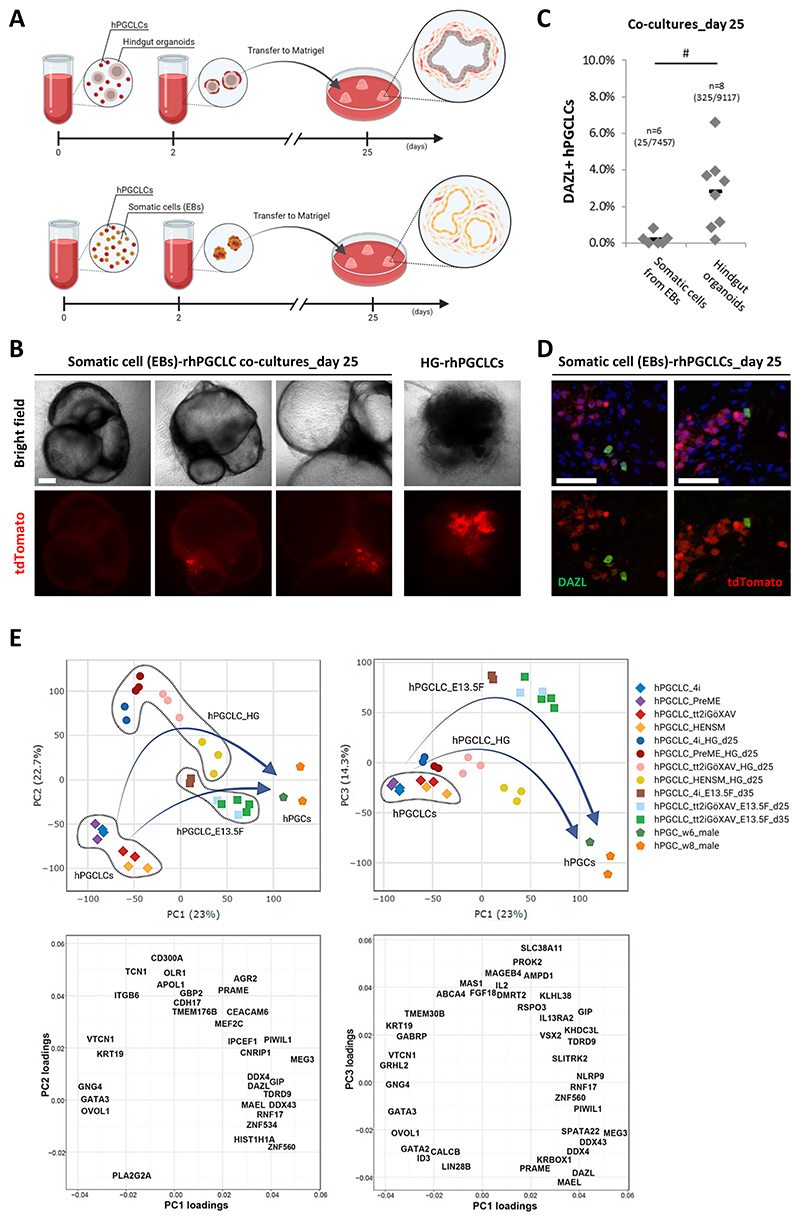
Progression of resetting hPGCLCs supported by human hindgut organoid and mouse female gonadal somatic cell co-cultures (A) Schematic diagram for the co-culture strategy of hPGCLCs with human hindgut organoids or somatic cells from embryoid bodies (EBs; negative control). (B) Co-culture of human hindgut organoid (HG) or somatic cells from embryoid bodies (EBs; negative control) with NANOS3−tdTomato resetting (tt2iGöXAV) hPGCLCs over the 25-day period. Scale bar, 500 um. (C) Percentage of DAZL positive resetting (tt2iGöXAV) hPGCLCs out of NANOS3-tdTomato positive hPGCLCs in day 25 co-cultures with human hindgut organoids or somatic cells from embryoid bodies (EBs; negative control). Horizontal bars represent the mean percentage for each condition. At least n=6 measurements were taken from independent experiments for each condition. Total number of cells counted (double DAZL and tdTomato positive/tdTomato positive cells) per condition shown between brackets. T-test: ^#^p-value <0.05. (D) Immunofluorescence of DAZL on sections of co-cultures of somatic cells from embryoid bodies with NANOS3−tdTomato resetting (tt2iGöXAV) hPGCLCs for 25 days. DAPI nuclear counterstain showed in blue. Scale bar, 50 um. (E) Two-dimensional principal component analysis (PC1 vs PC2 and PC1 vs PC3) and the respective gene loading plots for male weeks (w) 6 and 8 hPGCs, day 4 hPGCLCs, and hPGCLCs co-cultured with human hindgut organoids (HG) or mouse female gonadal somatic cells (E13.5F). Duration of co-cultures in days (d) is indicated. hPGCLCs were specified from peri-gastrulation (4i and PreME), resetting tt2iGöXAV, or resetting HENSM precursors. Arrows show the potential progression trajectories. See also Figures S3, S4, S11, and S12.

The supportive role of HG co-cultures for rhPGCLCs progression was validated by co-culturing rhPGCLCs with somatic cells from their originating embryoid bodies, as a negative control, in the same conditions as with HGs (Figure 4A). In general, somatic cells from embryoid bodies poorly supported rhPGCLCs during the co-culture period (Figure 4B). For the rhPGCLCs that persisted for 25 days in the negative control conditions (Figure 4B), DAZL expression was detected in approximately 0.2% of these cells (Figure 4C and 4D); but these were fewer than rhPGCLCs co-cultured with HGs (Figure 4C).

#### Co-culture with mouse embryonic ovarian cells

To further substantiate the results obtained with the HG cultures, we co-cultured rhPGCLCs (tt2iGöXAV) or peri-gastrulation (4i) hPGCLCs with E13.5 mouse ovarian somatic cells (E13.5F), adapting a previously published protocol^40,41^ (Figure S12A). After removing the endogenous mouse PGCs (see Methods, Figure S12B and S12C), we combined E13.5F ovarian somatic cells with hPGCLCs in ultra-low attachment 96-wells for two days (Figure S12D and S12E). After aggregation, cell clumps were placed on agarose stands and cultured for an additional 33 days in the air-liquid interface, as described previously^42^ (Figure S12E). Consistent with our findings in the hindgut co-cultures, we detect the expression of DAZL in approximately 2% of the co-cultured rhPGCLCs, which was not observed in peri-gastrulation hPGCLCs (Figure S12F and S12H). We also observed DDX4-positive rhPGCLCs in E13.5F ovarian somatic cell co-cultures (Figure S12G) and DAZL-positive rhPGCLCs with low levels of 5mC (Figure S12I).

#### Transcriptomic analysis on rhPGCLC progression

Next, we sequenced and integrated the transcriptome of resetting and peri-gastrulation hPGCLCs co-cultured with HG or E13.5F ovarian somatic cells, as well as weeks 6 and 8 hPGCs. Overall, both resetting (tt2iGöXAV and HENSM) and peri-gastrulation (4i and PreME) co-cultured hPGCLCs progress along PC1 from newly specified hPGCLCs towards the transcriptomic profiles of weeks 6 and 8 hPGCs (Figure 4E). Remarkably, rhPGCLCs progress further than peri-gastrulation hPGCLCs irrespective of co-cultured with either HG or E13.5F ovarian somatic cells (Figure 4E), demonstrating the higher capability of rhPGCLCs to progress in vitro. Specifically, we identified a set of 130 deferentially expressed genes upregulated in rhPGCLCs co-cultured with HG (e.g. *DAZL, DDX4, MAEL*, and *ZNF98*), which are linked to hPGC progression (Figure S11G and Figure S4). In addition to the support provided for hPGCLC progression (PC1), HGs and E13.5F ovarian somatic cells induce environment-specific transcriptomic changes to the co-cultured hPGCLCs. More specifically, genes strongly upregulated in hPGCLCs co-cultured with HG (e.g. *TCN1, OLR1*, and *PRAME*) contributed to their segregation along PC2, while genes prominently upregulated in hPGCLCs co-cultured with E13.5F ovarian somatic cells (e.g. *GIP, PROK2*, and *RSPO3*) contributed to their separation on PC3 (Figure 4E and Figure S4).

### EOMES is dispensable for rhPGCLC specification

Peri-gastrulation precursors express EOMES^14,15,17^(Figure S4A), which was reported to be an important transcription factor for the up-regulation of SOX17 and consequent hPGCLC specification^34^. Notably, EOMES expression was reduced in resetting relative to peri-gastrulation precursors raising the question of whether EOMES is essential for rhPGCLC specification (Figure 1H and I, and Figure S4A).

**Figure 5:**
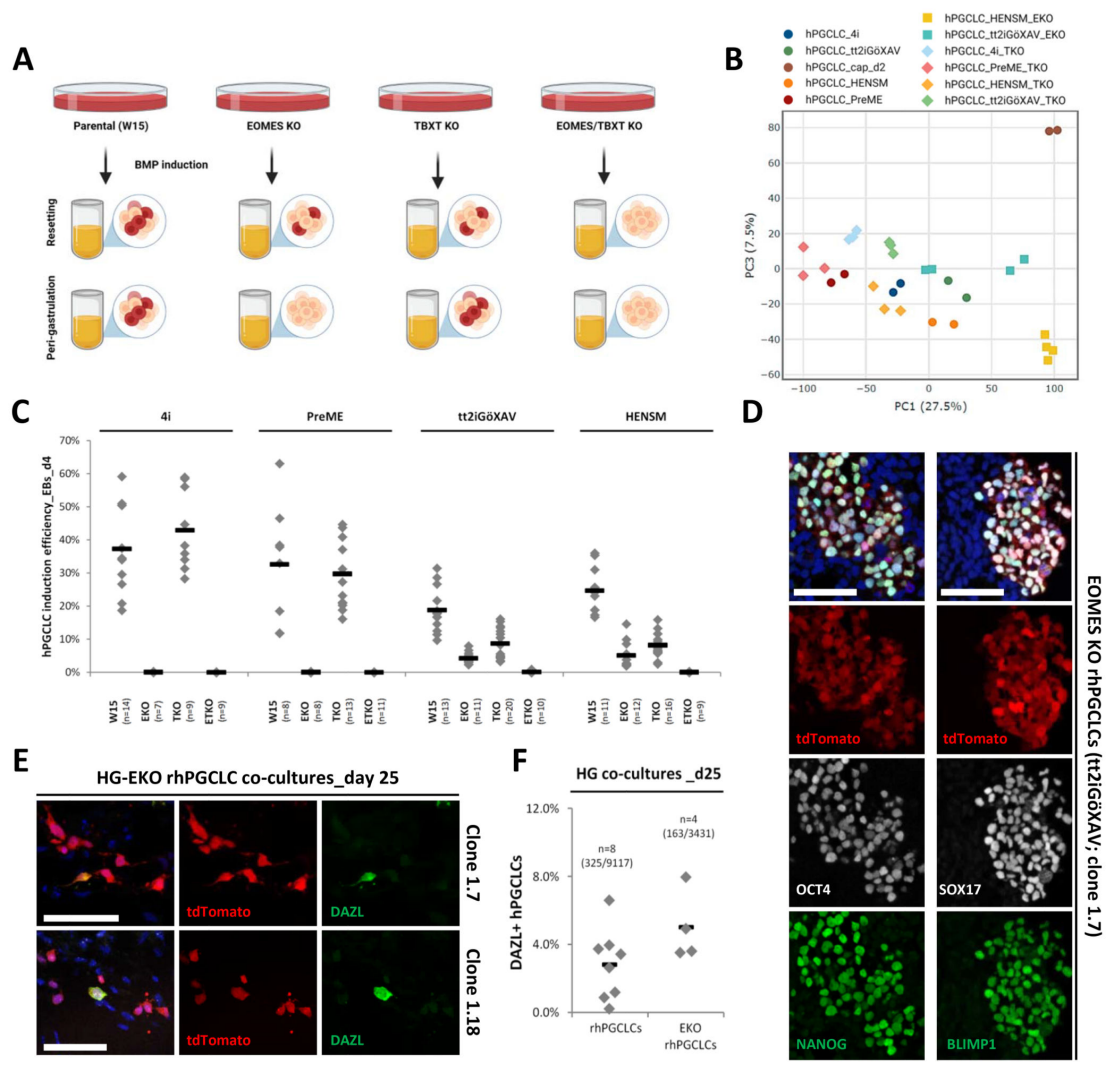
EOMES and TBXT requirements for resetting hPGCLC specification (A) Schematic diagram for hPGCLC specification experiments from EOMES, TBXT, and EOMES/TBXT knockout (KO) peri-gastrulation (4i and PreME) and resetting (tt2iGöXAV and HENSM) precursors. (B) Two-dimensional principal component analysis (PC1 vs PC2) for wildtype, EOMES knockout (KO), and TBXT knockout (TKO) day 4 hPGCLCs specified from peri-gastrulation (4i and PreME), resetting tt2iGöXAV, resetting HENSM, or capacitating (cap) precursors. (C) Efficiency of hPGCLC induction (% of hPGCLCs in day 4 embryoid bodies) from peri-gastrulation (4i and PreME) and resetting (tt2iGöXAV and HENSM) precursors for four genetic backgrounds (parental (W15), EOMES (EKO), TBXT (TKO), and EOMES/TBXT (ETKO) knockouts), measured by the co-expression of NANOS3−tdTomato and TNAP on flow cytometry analysis. Horizontal bars represent the mean percentage for condition. At least n=7 measurements were taken from independent experiments and for each condition. (D) Immunofluorescence of OCT4, SOX17, NANOG and BLIMP1 on sections from day 4 embryoid bodies containing rhPGCLCs expressing NANOS3−tdTomato specified from EOMES knockout (KO) resetting hESCs (tt2iGöXAV, clone 1.7). DAPI nuclear counterstain showed in blue. Scale bar, 50 um. (E) Immunofluorescence of DAZL on sections from day 25 human hindgut organoid (HG) co-cultures containing rhPGCLCs expressing NANOS3−tdTomato specified from EOMES knockout (EKO) resetting hESCs (tt2iGöXAV, clones 1.7 and 1.18). DAPI nuclear counterstain showed in blue. Scale bar, 50 um. (F) Percentage of DAZL positive tt2iGöXAV rhPGCLCs [wildtype and EOMES knockout (EKO)] out of NANOS3-tdTomato positive hPGCLCs in day 25 human hindgut organoid (HG) co-cultures. Horizontal bars represent the mean percentage for each condition. At least n=4 measurements were taken from independent experiments and for each condition. Total number of cell counted (double DAZL and tdTomato positive/tdTomato positive cells) per condition shown between brackets. See also Figures S3, S4, S13, S14, S15, and S16.

To test this hypothesis, we used CRISPR-Cas9 technology to generate EOMES knockout lines (EKOs) in the NANOS3−tdTomato reporter background (See Methods; Figure 5A). We obtained EKO lines with frameshift mutations resulting in a premature stop codon. All EKO lines exhibited similar morphology to their parental line when cultured in primed (E8), resetting (tt2iGöXAV and HENSM), and peri-gastrulation (4i and PreME) conditions (Figure S13A). Expression of EOMES was not detected in any of the EKO lines upon induction of mesodermal differentiation, in contrast to their parental line (W15, Figure S13B). Upon BMP induction, we observed hPGCLC specification from EKO resetting precursors, but not from EKO peri-gastrulation precursors (Figure 5C, Figure S14A, and Figure S15A). Although the induction efficiency was reduced to 20−25% of that obtained from the respective parental lines (Figure 5C), EKO rhPGCLCs expressed the hPGC key markers SOX17, BLIMP1, NANOG, and OCT4 (Figure 5D, and Figure S16A), as well as NANOS3−tdTomato and TNAP (Figure S15A). Notably, the overall transcriptome of EKO rhPGCLCs was very similar to rhPGCLCs (Figure S16B), with both hPGCLC types clustering closely together on PC analysis (Figure 5B).

Next, we tested the progression propensity of EKO rhPGCLCs (tt2iGöXAV) on co-cultures of either HGs or E13.5F ovarian somatic cells. We observed that EKO rhPGCLCs could be maintained for 25 days in HG co-cultures, where these were distributed in a scattered pattern similar to what was observed for EOMES wildtype rhPGCLCs (Figure 5E and Figure S16C). We also found that approximately 5% of the HG co-cultured EKO rhPGCLCs expressed DAZL (Figure 5E and 5F). Co-cultures with E13.5F ovarian somatic cells could also maintain EKO rhPGCLCs for 35 days, similar to EOMES wildtype rhPGCLCs (Figure S16D). DAZL expression was observed in approximately 1.0% of EKO rhPGCLCs co-cultured with E13.5F ovarian somatic cells for 35 days (Figure S16E and S16F). Together, these results show that EOMES is dispensable for both rhPGCLC specification and in vitro progression.

### Specification of rhPGCLCs requires both TBXT and EOMES

Next, we aimed to understand how rhPGCLCs could be specified in the absence of EOMES. We noticed that the expression of TBXT, another T-box transcription factor, increases during the conversion of hESCs from primed to naïve state, although at lower levels than observed in the conversion from primed to peri-gastrulation precursors (Figure S4A). Moreover, in mice, EOMES and T have overlapping and synergistic functions in controlling the initiation of gastrulation^43^, and are required for mPGC and mPGCLC specification^5,44^.

To evaluate whether TBXT is required for normal rhPGCLC specification and under EKO conditions, we deleted *TBXT* from both parental (W15) and EKO NANOS3−tdTomato reporter hESC lines using CRISPR-Cas9 technology (See Methods; Figure 5A). We obtained TBXT knockout (TKO) and EOMES/TBXT double knockout (ETKO) lines with frameshift mutations resulting in a premature stop codon. Both TKO and ETKO hESCs exhibited similar morphologies to their parental lines when cultured in primed (E8), resetting (tt2iGöXAV and HENSM) and peri-gastrulation (4i and PreME) conditions (Figure S13A). Expression of TBXT was not detected in both TKO and ETKO hESCs after differentiation into the mesoderm fate (Figure S13B). We observed hPGCLC specification from TKO resetting precursors, though it was reduced to 33−46% of the induction efficiency obtained from the parental lines. In line with previous findings^34^, the absence of TBXT did not affect the efficiency of hPGCLC induction from the peri-gastrulation precursors (Figure 5C, Figure S15A, and Figure S15A). Importantly, the transcriptome of TKO hPGCLCs specified from both resetting and peri-gastrulation precursors were very similar to the corresponding parental hPGCLCs (Figure S15B and Figure 5B), as demonstrated for EKO rhPGCLCs.

**Figure 6:**
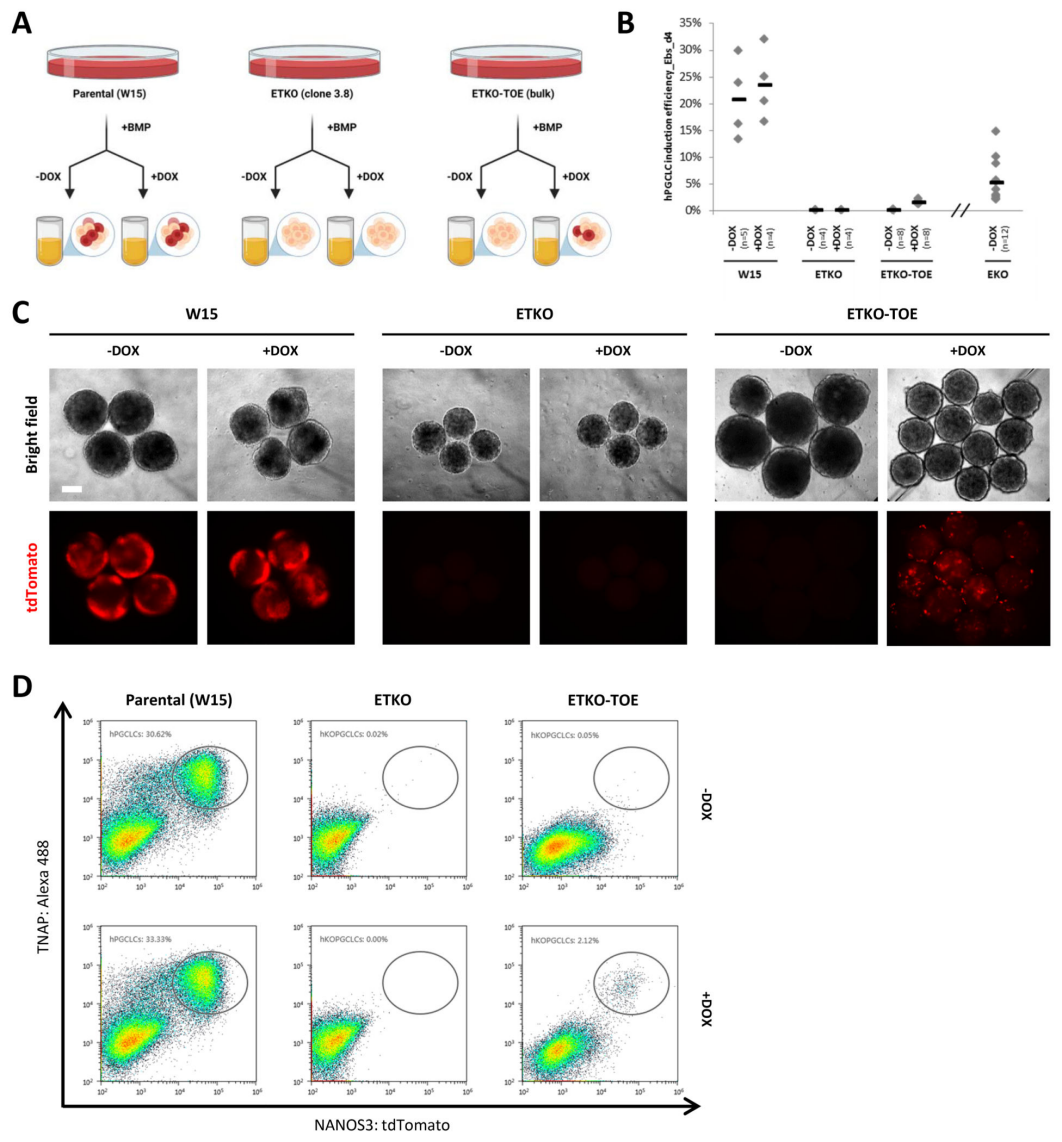
Contribution of TBXT over expression to rescue EOMES/TBXT double knockout phenotype in resetting hPGCLC specification (A) Schematic diagram for TBXT rescue experiment. (B) Efficiency of hPGCLC induction (% of hPGCLCs in day 4 embryoid bodies) from HENSM resetting hESCS in three different genetic backgrounds (parental (W15), EOMES/TBXT double knockout (ETKO), and DOX-inducible *TBXT* transgene on an ETKO background (ETKO-TOE)), under the absence or presence of DOX, measured by the co-expression of NANOS3−tdTomato and TNAP on flow cytometry analysis. Efficiency of hPGCLC induction from EOMES knockout (EKO) HENSM resetting hESCS, in the absence of DOX is also included (data from Figure 5C). Horizontal bars represent the mean percentage for each condition. At least n=4 measurements were taken from independent experiments and for each condition. (C) Day 4 embryoid bodies generated from parental (W15), EOMES/TBXT double knockout (ETKO), and DOX-inducible *TBXT* transgene on an ETKO background (ETKO-TOE) NANOS3−tdTomato hESCs cultured in resetting (HENSM) conditions, under the absence or presence of DOX. Scale bar: 200 um. (D) Flow cytometry analysis plots showing the percentage of hPGCLCs co-expressing NANOS3−tdTomato and TNAP in day 4 embryoid bodies generated from HENSM resetting hESCS in three different genetic backgrounds (parental (W15), EOMES/TBXT double knockout (ETKO), and DOX-inducible *TBXT* transgene on an ETKO background (ETKO-TOE)), under the absence or presence of DOX.

Notably, the deletion of both EOMES and TBXT (ETKO) led to the absence of hPGCLC specification from both resetting and peri-gastrulation precursors (Figure 5C, Figure S14A, and Figure S15A), suggesting that TBXT plays a crucial role in rhPGCLC specification in the absence of EOMES. To further corroborate these findings, we engineered a DOX-inducible *TBXT* transgene on an ETKO background (ETKO-TOE), which we utilized to test the overexpression of TBXT during rhPGCLC specification (Methods, Figure 6A). In the presence of DOX, rhPGCLC specification was partially rescued in ETKO-TOE resetting (HENSM) precursors (Figure 6B−D), demonstrating that the loss of rhPGCLC specification in the ETKO background is specific to the loss of TBXT. These observations suggest that rhPGCLC specification occurs under the combined influence of TBXT and EOMES (Figure 5C). This is in contrast to hPGCLC specification from peri-gastrulation precursors, where EOMES is essential and where TBXT cannot compensate for the absence of EOMES^34^.

## Discussion

Established approaches to reset primed hPSCs ^22,23^ and to capacitate naïve^26,27^ hPSCs allow the emergence of transient cell populations with transcriptomic profiles positioned between primed and naïve pluripotent states^25–27^ (Figure 1H and 1I). These transitioning populations acquire transcriptomic signatures that align with human and cynomolgus monkey early post-implantation epiblast states^24,26,27^. The resetting and capacitating states of pluripotency bear similarities to formative pluripotency^45^ proposed for early post-implantation mPGC-competent epiblast in E5.5−6.0 mouse embryos^1^ as well as for mPGCLC-competent mEpiLCs ^4,18^. Here, we show the specification of hPGCLCs from resetting and capacitating precursors, suggesting similarities with the mouse^4^ and rat^46^ models, as well as the early post-implantation cynomolgus monkey epiblast, competent for cyPGC specification in vivo^30,47^ and ex vivo^48,49^.

Although sharing the commitment to the germline fate, rhPGCLCs and peri-gastrulation hPGCLCs diverge in the levels of some epigenetic markers and in the expression of a restricted set of genes. Among these, some are maintained from the transcriptomic signatures of their precursors. These precursor-specific “inherited” signatures may also apply for some features of their epigenetic and metabolic profiles, which together might contribute to the differences observed in progression propensity. Interestingly, peri-gastrulation hPGCLCs express higher levels of somatic genes (e.g. amnion related genes) compared with rhPGCLCs. This might be a consequence of either the initial activation of somatic transcriptional programmes in peri-gastrulation precursors or the increased susceptibility of these precursors to activate somatic programmes under hPGCLC specification conditions. The delay in suppressing these somatic programmes may slow the progression of peri-gastrulation hPGCLCs. On the other hand, resetting precursors have transcriptomic profiles between naïve and primed pluripotency and consequently less somatic transcriptional activity. Accordingly, rhPGCLCs not only express low levels of somatic-related genes, but also start expressing genes related to hPGC progression. Altogether, while peri-gastrulation hPGCLCs probably represent pre-migratory hPGCs^13,17^, rhPGCLCs are developmentally more advanced as observed for mouse^4,13^ and rat^46^ PGCLCs, which are considered to represent early migratory PGCs.

In ex-vivo embryos, hPGCs are reported in E12−E17 embryos^7,9^. The heterogeneous expression of progression genes, including DAZL, starts 17−23 days after, in E29−E35 (CS12−14) embryos, during migration through the hindgut and dorsal mesentery^38^ (Figure 3F). Accordingly, 25-day co-culture with hindgut organoids led to the heterogeneous up-regulation of hPGC progression genes in rhPGCLCs, which closely resembles the timing observed in vivo. Notably, the progression of rhPGCLCs is not only linked to their higher intrinsic competency but also associated with the support provided by the human hindgut organoids co-cultures, which were expected to represent a more physiological and allogenic environment, equivalent to the migration of hPGCs in vivo. In line with our observations, these organoids were recently demonstrated to have a robust capacity to induce maturation of other cell lineages^50^.

Progression of hPGCLCs specified from the peri-gastrulation iMeLC precursors has recently been reported^40,51^. In these studies, hPGCLCs were co-cultured for up to 120 days with somatic cells from either female^40^ or male^51^ mouse embryonic gonads, providing proof of concept for the in vitro progression potential of hPGCLCs. In these studies, the expression of DAZL in iMeLC-derived hPGCLCs was only observed after 77 days of co-culture^40,51^. Using the same culture principles, we showed that rhPGCLCs start expressing DAZL and other progression markers after only 35 days in culture, validating the higher capability of rhPGCLCs for progression. In future experiments, the isolation of progressed rhPGCLCs from the hindgut organoid co-cultures and subsequent combination with human gonadal somatic cells might recapitulate the stepwise in vivo progression of hPGCs more faithfully and demonstrate the cumulative and instructive cues experienced by hPGCs on their route to the gonads.

To further highlight the differences between resetting and peri-gastrulation hPGCLCs, we focused on their precursors. Recent in vivo^30^ and ex vivo^48,49^ studies identified putative cyPGCs and cyPGCLCs on cynomolgus monkey early post-implantation embryos with no detectable levels of EOMES in their epiblast cells. This, together with the fact that *EOMES* expression was not detected in resetting precursors (Figure S4A), made us consider that the role of EOMES may diverge between the hPGCLC-competent resetting and peri-gastrulation precursors. Collectively, our observations establish that EOMES is partially dispensable to initiate the rhPGCLC specification program from resetting precursors, which is not the case for peri-gastrulation precursors where EOMES is either necessary to reach the competent state and/or to initiate hPGCLC specification from that point on^34^.

In the mouse, EOMES and T have overlapping roles in controlling pluripotency exit and germ-layer segregation during gastrulation^43^. Moreover, both EOMES^44^ and T^5^ are singularly necessary for mouse PGC and PGCLC specification, suggesting a cooperative function for this process. In addition, T/TBXT expression has been reported in mouse^5^, pig^15^ and cynomolgus monkey^30^ PGC precursors. We also noticed the expression of *TBXT* in resetting precursors, although to lower levels than those observed in peri-gastrulation precursors (Figure S4A). Together, these observations led us to test the role of TBXT for rhPGCLC specification from EKO resetting precursors. Our results established that, in contrast to peri-gastrulation precursors, rhPGCLC specification from resetting precursors depends on both EOMES and TBXT, highlighting yet another critical difference between peri-gastrulation and resetting precursors.

An intriguing question is why TBXT cannot compensate for the absence of EOMES during hPGCLC specification from peri-gastrulation precursors. TBXT and EOMES belong to the T-box family of transcription factors, which binds to the consensus motif TTCACACCT ^52,53^. Since different T-box members exhibit preferences for the number, spacing and orientation of the consensus motif whilst tolerating some variation in its sequence^52,53^, it is entirely plausible that the same motifs can be used by several T-box members. The affinity of T-box members for their binding motifs can vary along developmental or differentiation paths due to epigenetic changes or co-factor availability, suggesting context-dependency for their transcriptional activation roles^54^. Therefore whilst TBXT may be able to compensate for the absence of EOMES in common (such as *SOX17*) or TBXT-specific targets during hPGCLC induction from resetting precursors, the function of TBXT on these common or specific targets might be reduced in the context of hPGCLC induction from peri-gastrulation precursors, thus insufficient to rescue hPGCLC specification.

**Figure 7:**
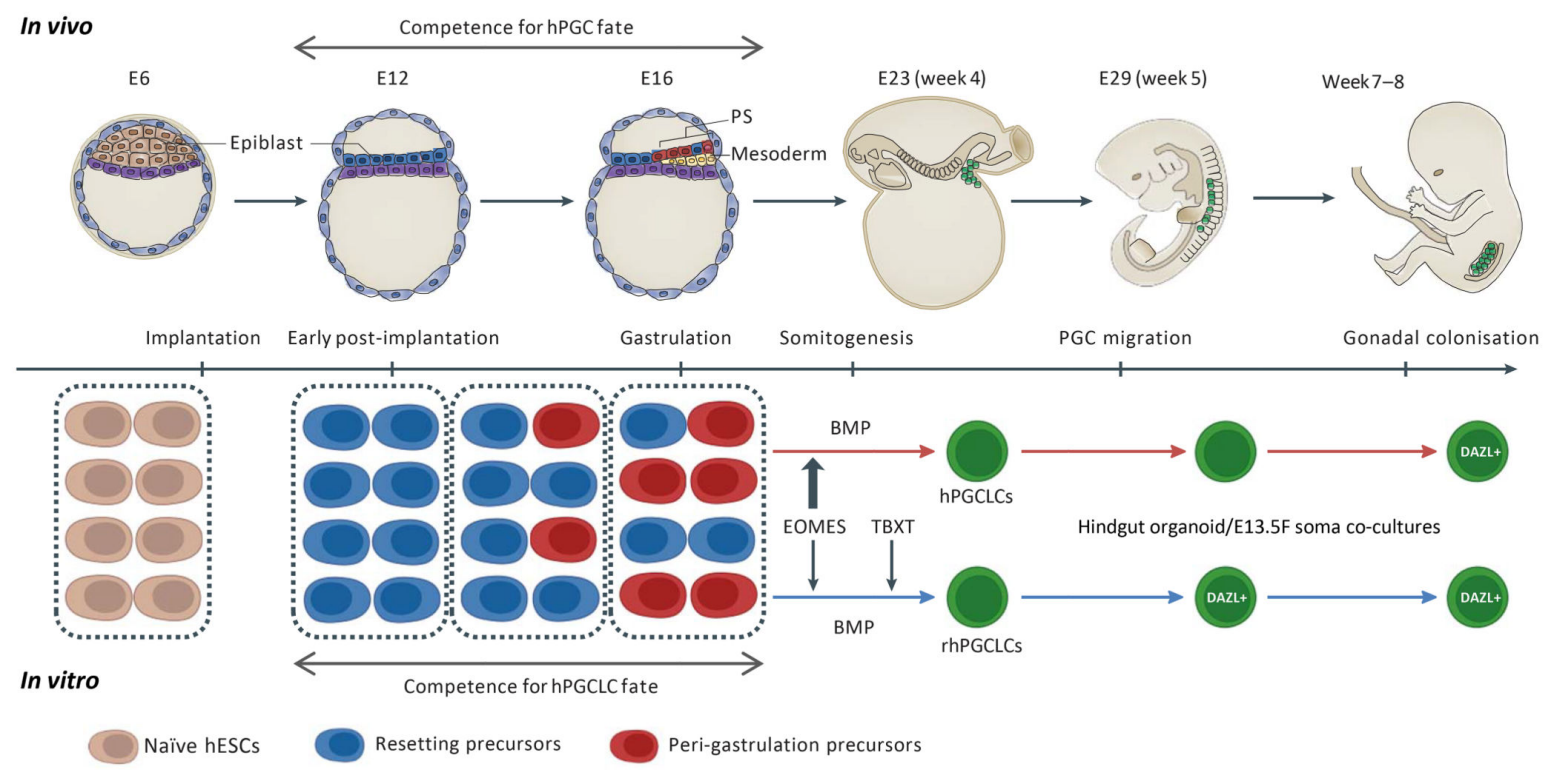
Schematic diagram for in vitro protocols of hPGCLC specification and in vivo hPGC development. Double headed arrows delineate the edges of the spectrum for hPGCLC specification in vitro and suggest a window for hPGC specification in vivo (E11-E17). The three blocks of cells represent the possibility of a temporally asynchronous epiblast, constituted by a decreasing ratio of early post-implantation epiblast precursors (modelled by in vitro resetting cells) and an increased ratio of peri-gastrulation precursors (modelled by in vitro peri-gastrulation cells), along early post implantation developmental time. Resetting hPGCLCs specification requires both TBXT and EOMES, while peri-gastrulation hPGCLCs rely exclusively on EOMES to be specified. Resetting hPGCLCs progress faster than peri-gastrulation hPGCLCs, and at a tempo similar to that observed in vivo. Elements of the diagram were adapted from Tang et al, 2016 ^13^.

Our results highlight that distinct hPGCLC precursors bearing hallmarks of different stages of post-implantation epiblast exist in vitro, which may be the case *in vivo*. Two scenarios may explain an extended window of hPGC competence in the post-implantation epiblast. In the first scenario, epiblast cells could retain the plasticity to specify hPGCs throughout early post-implantation to the onset of gastrulation while its transcriptomic identity changes correspondingly through developmental time. The alternative is the possibility of a temporally asynchronous epiblast (Figure 7A). This scenario is supported by recent observations showing the existence of heterogeneous cell profiles in the early rabbit^55^ and human^56^ epiblasts with overlapping cellular identities in consecutive developmental stages^56^. In this situation, hPGC-competent epiblast cells that maintained an early post-implantation state in a peri-gastrulation staged embryo may respond to the same hPGC specifying cues as other hPGC-competent epiblast cells that have acquired peri-gastrulation state (Figure 7A). Our results show that rhPGCLC precursors bear hallmarks of early human post-implantation epiblast, which in vivo might be maintained in some epiblast cells throughout early post-implantation to the onset of gastrulation^56^. However, further testing will be required on cultured human embryos to determine the identity of precursors during the entire window of hPGC specification.

In conclusion, we demonstrated the specification of hPGCLCs from resetting precursors, which showed an enhanced capability to progress in vitro. The progression of these cells was supported by human hindgut co-cultures and occurred at a tempo similar to that observed in vivo. Importantly, we demonstrated that resetting precursors have a distinctive transcriptomic identity and that these cells rely on both EOMES and TBXT to specify rhPGCLCs. Although the exact identity of hPGC precursors remains unknown, it is possible that competent epiblast precursors with distinct identities might converge to the hPGC fate, as described for other cell lineages^57^. In vitro, our resetting precursors and their derived rhPGCLCs appear as promising systems to model hPGC specification and development, paving the way for developing efficient protocols for hPGCLC progression. Importantly, these in vitro models create opportunities to study human germline-associated diseases, including human infertility, and therapeutic approaches to minimize their impact on human health.

### Limitations of the Study

Although we were privileged in having access to rare human embryonic samples described here, we only analysed one sample per developmental stage. Future analysis of additional samples will be needed to strengthen the observations made here. While we have demonstrated here that hindgut organoids can support hPGCLC progression, we did not perform a comprehensive characterization of these organoids and their interactions with hPGCLCs. In future, it will be interesting to molecularly compare this co-culture system with ex vivo human embryonic hindgut and migratory hPGCs in order to improve the culture conditions described here.

## STAR Methods

### Resource Availability

#### Lead Contact

Further information and requests for resources and reagents should be directed to João Pedro Alves-Lopes (joao.pedro.lopes@ki.se).

#### Materials Availability

There are restrictions to the availability of human lines generated in this study due to constraints in the Materials Transfer Agreement of their parental lines.

### Experimental Model And Subject Details

#### Mouse embryonic tissue samples

We used female E13.5 mouse embryos carrying the Oct4ΔPE-GFP transgene (MGI:3693125). All experimental procedures were carried out in agreement with the project licence PE596D1FE issued by the UK Home Office and carried out in a Home Office designated facility. In particular, mice were maintained with a 12 hour light/12 hour dark cycle, with temperature ranging from 20-24°C and humidity of 45-65%. Mice were housed under standard husbandry conditions and under UK Home Office and institute regulations, with four to five mice housed per cage, and with free access to water and food. Matings were performed with males and females greater than 10 weeks of age. E13.5 embryo samples were taken from ethically euthanized pregnant females following timed mattings.

#### Human embryonic tissue samples

Human embryonic tissues were collected from medical or surgical terminated embryos at Addenbrooke’s Hospital (Cambridge, UK) after donor consent and authorization from NHS Research Ethical Committee (REC No: 96/085). The developmental stage of the collected embryos was estimated by crown-rump length and anatomical features (e.g., limbs and digits morphology) referenced to the Carnegie staging (CS). In addition, lower embryonic bodies of CS12 and CS13 samples were collected and staged by the Human Developmental Biology Resource (HDBR)-UCL (Project 200421) with ethics approval and patient consent forms held under the UCL branch (REC: 18/LO/0822). All samples were handled and stored according to the Human Tissue Act (HTA) regulations, and following Gurdon Institute Safety-committee approved Risk Assessments and Procedures. For all samples, embryo sex was revealed by sex genotyping PCR as defined before^11,17^.

#### Human ESCs

The use of hESCs in our study was approved by the Human Biology Research Ethics Committee from the University of Cambridge (HBREC.2019.06).

Primed hESCs WIS2 (P33), W24 (P44), and W15 (P54) were maintained in Essential 8 medium (E8; Thermo Fisher Scientific) on vitronectin (Thermo Fisher Scientific) coated wells and passaged in small cell clumps every 3−4 days with 0.5 mM EDTA (Thermo Fisher Scientific) in PBS.

### Method Details

#### Culture conditions for hESCs

When necessary, primed hESCs were directly converted into peri-gastrulation hPGCLC-competent 4ihESCs, as described previously^17^. Briefly, primed hESCs were dissociated with TrypLE express (Thermo Fisher Scientific) and seeded on irradiated mouse embryonic fibroblasts (MEFs; Insight Biotechnology) in knockout DMEM (Thermo Fisher Scientific) supplemented with 20% (vol/vol) knockout serum replacement (Thermo Fisher Scientific), 2 mM L-glutamine (Thermo Fisher Scientific), 0.1 mM Non-Essential Amino Acids (NEAA; Thermo Fisher Scientific), 0.1 mM 2-mercaptoethanol (Thermo Fisher Scientific), 1% (vol/vol) penicillin/streptomycin (PenStrep; Thermo Fisher Scientific), 20 ng/ml human LIF (hLIF; Biochemistry Department, University of Cambridge (BD-UCAM)), 8 ng/ml bFGF (BD-UCAM), 1 ng/ml TGF-β1 (Peprotech), 3 μM GSK-3i CHIR99021 (GSK-3i; Tocris), 1 μM MEK inhibitor PD0325901 (MEKi; Tocris), 5 μM p38 MAPK inhibitor SB203580 (Tocris), 5 μM JNK inhibitor SP600125 (Tocris) and 10 μM of ROCK inhibitor Y27632 (ROCKi; Tocris), for the first 24 h. Afterwards, 4ihESCs were cultured with the same medium without ROCKi and passaged every 3−4 days using TrypLE express.

Another peri-gastrulation hPGCLC-competent state was obtained by differentiating hESCs into mesendodermal precursors (preME), as described previously^15^. Briefly, primed hESCs were dissociated into single cells, seeded on vitronectin coated wells (200,000 cells per well of a 12-well plate) and incubated in Advanced RPMI 1640 medium (Thermo Fisher Scientific) supplemented with 1% (vol/vol) B-27 supplement (Thermo Fisher Scientific), 0.1 mM NEAA, 1% (vol/vol) PenStrep, 2 mM L-glutamine (aRB27 basal medium), 100 ng/ml activin A (BD-UCAM), 3 μM GSK-3i and 10 μM of ROCKi for 12h at 37 °C in 5% CO_2_.

In order to obtain resetting precursors, primed hESCs were reset towards a state of naïve pluripotency by two previously described protocols (tt2iGöXAV and HENSM) ^22,33^. In a first approach, we used the stepwise conversion protocol described by Guo et al. (tt2iGöXAV)^22^. Briefly, primed hESCs were dissociated into single cells and seeded on irradiated MEFs in E8 medium supplemented with 10 μM of ROCKi at 37 °C in 5% CO_2_ and atmospheric O_2_. After 24 h, we washed the cultures with PBS and added a 1:1 mixture of DMEM/F-12 (Thermo Fisher Scientific) and Neurobasal (Thermo Fisher Scientific) media supplemented with 0.1 mM NEAA, 2 mM L-glutamine, 1% (vol/vol) B-27 supplement, 0,5% (vol/vol) N-2 supplement (Thermo Fisher Scientific), 0.1 mM 2-mercaptoethanol (N2B27 medium), 10 ng/ml hLIF, 1 μM MEKi PD0325901 and 1 mM Valproic acid (Alpha Laboratories Ltd.). The medium was changed every day and, from there on, cells were incubated at 37 °C in 5% CO_2_ and 5% O_2_. After 3 days, we washed the cultures with PBS and replaced medium with N2B27 supplemented with 10 ng/ml hLIF, 1 μM MEKi PD0325901, 2 μM PKCi Gö6983 (PKCi; Abcam), 2 μM TNKi XAV939 (TNKi; Sigma) and 2 μM WNTi IWP2 (Tocris). The medium was changed every day, for the next 5 days. After, cells were dissociated with Accutase (Thermo Fisher Scientific) and seeded on Matrigel (Thermo Fisher Scientific) coated wells in N2B27 medium supplemented with 10 ng/ml hLIF, 1 μM MEKi PD0325901, 2 μM PKCi, 0.3 μM GSK-3i and 2 μM TNKi, as for the following passages. Resetting and fully reset tt2iGöXAV hESCs were passaged every 5−6 days, up to P20.

In a second approach to obtain resetting precursors, we utilized a direct resetting conversion protocol optimized by Bayerl et al. (HENSM)^33^. Concisely, primed hESCs were dissociated into single cells and seeded on Matrigel-coated wells in a 1:1 mixture of Neurobasal and DMEM/F-12 media supplemented with 1% N-2 supplement, 2 mM L-glutamine, 0.1 mM NEAA, 100 U/ml penicillin, 1% (vol/vol) PenStrep, 2% (vol/vol) B-27 supplement, 0.8mM Dimethyl 2-oxoglutarate (Sigma), 0.2% Matrigel, 50μg/ml L-Ascorbic acid 2-phosphate sesquimagnesium salt hydrate (Sigma), 20 ng/ml hLIF, 1 μM MEKi PD0325901, 2 μM TNKi, 0.8 μM P38i/JNKi BIRB0796 (Axon Medchem), 2 μM PKCi Gö6983, 1.2 μM ROCKi, 1.2μM SRCi CGP77675 (Axon Medchem) and 10 ng/ml recombinant human activin A. Medium was changed every day and cells were cultured at 37 °C in 5% CO_2_ and 5% O_2_. Resetting HENSM hESCs were passaged every 5−6 days, up to P10.

To obtain capacitating hESCs, we capacitated fully reset/naïve tt2iGöXAV hESCs (P10-P20) towards primed pluripotency using a protocol described by Rostovskaya et al. (N2B27XAV)^26^. Briefly, naïve tt2iGöXAV hESCs were dissociated into single cells with Accutase and seeded on Matrigel-coated wells in N2B27 medium supplemented with 2 μM TNKi and 10 μM of ROCKi. Medium without ROCKi was renewed every day and cells were cultured at 37 °C in 5% CO_2_ and atmospheric O_2_. Capacitating hESCs were cultured up to 6 days.

#### Generation of EOMES and TBXT knockout lines

CRISPR-Cas9 gene targeting technology was used by transfecting hESCs (W15) with an in vitro assembled ribonucleoprotein (RNP) containing the EnGen® Spy Cas9 NLS protein (20μM from NEB) and pre-designed 3x gRNAs targeting *EOMES* exon 2 or *TBXT* exon 1 coding regions (Gene KO Kit v2, Synthego). Reagents were used in accordance with the manufacturer’s instructions. In brief, 1.5 nmol of lyophilised gRNAs were dissolved in TE buffer to achieve a 200 μM stock. The RNP was assembled by combining 20 pmols of Cas9-NLS to 45 pmols of gRNA mix in a 1:1 volume ratio and incubated at room temperature for 20 minutes. In the meantime, low passage primed hESCs (W15, P54) were dissociated using TrypLE in a humidified 37°C incubator for 5 minutes followed by inactivation with DMEM/F-12 supplemented with 10% (vol/vol) FBS. hESCs (200,000 cells) were resuspended in 20 μL of complete P3 buffer (Lonza P3 Primary Cell 4D-Nucleofector Kit) with the assembled RNP complex and transferred to the provided 16-well strip electrodes. The RNP were delivered to hESCs using the 4D-nucleofector (Lonza) set to the P3 program CA-137. Cells were immediately transferred to room temperature E8 medium supplemented with 1.3% cell-culture grade fatty acid-free BSA (WAKO; E8BSA) and 10 μM ROCKi on a well of a 12-well plate pre-coated with 2% (vol/vol) Matrigel overnight. 24 hours after transfection, media was changed to E8 without supplementations with daily media changes.

Visibly separated colonies are manually picked under an inverted microscope (Zeiss Axio A1) after washing the well with PBS and adding 0.5mM EDTA/PBS. After RT incubation for 3-4 minutes, the tight association of cells were loosened but not detached. Picked colonies were placed in a well of a 96-well plate pre-coated with 2% (vol/vol) Matrigel containing E8BSA with 10 μM ROCKi and 1% (vol/vol) penicillin/streptomycin. This cell suspension was split into two wells of a 96-well plate after pipet mixing. 24 hours after plating, media was changed to E8 without supplementations with daily media changes. Confluent wells can be passaged as normal.

For genotyping, cells in one of the two wells were dissociated using TrypLE, neutralised with DMEM/F-12 with 10% (vol/vol) FBS, counted, centrifuged and re-suspended at 2000 cells/μL in a lysis/PCR buffer containing 400 μg/ml proteinase K (sigma) and 1x PCR buffer (KAPA). Cells were lysed at 56°C for 10 minutes and proteinase K is inactivated at 98°C for 10 minutes. 1 μL of this lysed cell solution can be used directly for high-fidelity PCR reaction (KAPA) using primers AAAGGGCCGAGAATATGAGCC (F) and GGGTGGGGTGATGTCTTAGTT (R) flanking the gRNA targeting region of *EOMES*. The PCR amplified band is gel excised (NEB Monarch) and submitted to sequencing using the same primers. Results were analysed using ICE (Synthego) to identify clones with knock-out frameshift mutations. We selected 4 (1.4, 1.7. 1.18 and 1.19) out of 45 picked clones.

As *TBXT* targeting was much less efficient in generating a frame-shift truncation due to the distance between the predesigned gRNAs, we sought to find clones that were devoid of detectable levels of TBXT protein. Primers CTGGGTCTGATATGGCCGCT (F) and GGTCGGGACACCGAAGTG (R) were used for the targeting region of *TBXT*. We found TBXT single knockout clone 1.1, among 23 picked clones. The validated EOMES single knockout line 1.7 (P54+3) was further targeted with aforementioned *TBXT* gRNAs to derive the TBXT/EOMES double knockout line 3.8, among 44 picked clones.

#### TBXT rescue line

As TBXT/EOMES double knockout line (ETKO 3.8) did not specify hPGCLCs in any conditions, TBXT rescue line (ETKO-TOE) was generated on top of clone ETKO 3.8 (P54+3+3) by transfecting a single polycistronic, insulated plasmid comprising doxycycline-responsive expression of GFP-T2A-TBXT and constitutively expressing rtTA (Tet3G)-*IRES*-Puro under the promoter EF1α. The plasmid was lipofected (Lipofectamine Stem, Invitrogen) into primed hESCs with simultaneous transient expression of piggyBac transposase (PBase) for stable integration. To take advantage of over-expression level heterogeneity, 8 independently transfected whole puro-resistant populations of cells were used for hPGCLC inductions and analysis.

#### SOX17 rescue line

A previously established NANOS3-mCherry SOX17 knockout line SKO5 (P47+4)^17^ was used to transfect two plasmids to derive the SOX17 rescue line S17.11. One plasmid carries a constitutively expressing rtTA(Tet3G)-*IRES*-Hygro under the CAG promoter, while the other plasmid contains doxycycline-responsive expression of *SOX17*. The plasmids were lipofected (Lipofectamine Stem, Invitrogen) into SKO5 hESCs with simultaneous transient expression of piggyBac transposase (PBase) for stable integration. To ensure successive propagation of unsilenced transgene expressing permissive level of exogeneous SOX17, hygromycin-resistant clones were selected and hPGCLC induction efficiency was tested over several passages to reach efficiencies near parental levels.

#### Genome integrity assessment

The hESC lines WIS2, W24, W15, and SOK5 have normal karyotypes, which were previously confirmed by G-banding karyotype analysis^15,17^.

For the hESC lines generated for this publication (S17.11, EKO 1.4, EKO 1.7, EKO 1.18, EKO 1.19, TKO 1.1, ETKO 3.8, and ETKO-TOE), G-banding karyotype analysis was performed at Clinical Genetics Department, Karolinska University Hospital. Normal karyotypes were confirmed in 20 out of 20 cells for all hESC lines analysed.

#### Human PGCLC specification

Peri-gastrulation hPGCLCs (4i or PreME) were obtained as described before^15,17^. Briefly, competent 4ihESCs or PreME precursors were dissociated into single cells by TrypLE express and collected into an ULA 96-wells (96-well ultra-low attachment plate; Corning) at the concentration of 4000 cells per well. Peri-gastrulation hPGCLCs were specified in aRB27 medium supplemented with 500 ng/ml BMP2 (BD-UCAM), 100 ng/ml SCF (Peprotech), 10 ng/ml hLIF, 50 ng/ml EGF (R&D Systems), and 10 μM ROCKi for 3−5 at 37 °C in 5% CO_2_ and atmospheric O_2_.

Resetting hPGCLCs (rhPGCLCs) were induced from early passages (P1−P5) of resetting hESC precursors (tt2iGöXAV and HENSM). Resetting hESCs were exposed to Accutase in order to render single cells and small cellular aggregates, which were collected into ULA 96-wells at the concentration of 4000 cells per well. The same induction medium and conditions were utilized as for peri-gastrulation hPGCLCs, however, we added 0.25% (v/v) poly-vinyl alcohol (Sigma) to support cell aggregation.

Capacitating hPGCLCs were induced from capacitating precursors (days 1−6). Capacitating hESCs were dissociated into single cells using Accutase and collected into ULA 96-wells at the concentration of 4000 cells per well. The same induction medium and conditions were utilized as for rhPGCLCs.

#### Human hindgut organoid specification

Human hindgut organoids were obtained by sequential differentiation of primed hESCs into mesoderm-like, endoderm-like and posterior endoderm-like tissues, following previously established protocols^36,37^ with few modifications. Concisely, confluent 12-wells of primed hESCs were dissociated and seeded on vitronectin coated wells of a 12-well plate, in mesoderm differentiating medium^15^, at 200,000 cells per well. Mesoderm differentiating medium is composed of arB27 medium supplemented with 3 μM of GSK-3i, 100 ng/ml of activin A, and 10 μM of ROCKi. After 24h, mesoderm medium was replaced with endoderm differentiation medium, consisting of arB27 medium supplemented 100 ng/ml of activin A and 0.5 μM of the BMP inhibitor LDN193189 (Sigma). Endoderm differentiation was carried out for 3 days and the medium changed every day. Ultimately, endoderm medium was replaced by posterior endoderm/hindgut differentiation medium that consists of arB27 medium supplemented with 3 μM of GSK-3i and 100 ng/ml of FGF4 (R&D Systems). Posterior endoderm differentiation was carried out for 4−5 days and the medium changed every day.

#### Human hindgut organoid and hPGCLC co-cultures

After posterior endoderm specification, small spherical hindgut organoids were floating or loosely attached to the bottom of the culture well. Still attached hindgut organoids were gently detached with the help of a needle. Three to five of these hindgut organoids were placed per ULA 96-well with 100 μL of arB27 medium supplemented with 100 ng/ml SCF, 25 ng/ml EGF (HG medium), and 10 μM of ROCKi (HG medium + ROCKi). Sorted hPGCLCs (approximately 2,000 cells) were suspended in 100 μL of HG medium + ROCKi and added to each of the previously prepared wells with hindgut organoids. Cells- and tissue-like structures were centrifuged for 2 minutes, at 1200 rpm and afterwards allowed to aggregate for 2 days at 37 °C. After, each aggregate was embedded in 15 μL of an extracellular matrix solution (2 parts of Matrigel and 1 part of HG medium), on the bottom of a 12-well plate. Up to 4 aggregates were cultured per well. Once the 15 μL-drops of Matrigel was gellified (approximately 20 minutes), 1 ml of HG medium was added to each 12-well. The hindgut organoid and hPGCLC co-cultures were incubated at 37 °C, in 5% CO_2_. Media was changed every 2 days.

#### Ovarian somatic cells collection from mouse embryos

Developing gonadal ridges were collected from embryonic day (E) 13.5 mouse embryos carrying the Oct4ΔPE-GFP transgene. After being dissected from the connected mesonephric tissue, ovaries were dissociated with 0.25% trypsin/EDTA (Thermo Fisher Scientific) into single cell suspensions. We took advantage of the OCT4ΔPE -GFP reporter to sort out GFP+ mPGCs using a Sony SH800 Cell Sorter. The mouse E13.5 ovarian somatic cell fraction (GFP-) was collected and either cryopreserved or freshly used in our downstream co-culture experiments.

#### Mouse ovarian somatic cells and hPGCLC co-cultures

We combined 1,500 hPGCLCs with 15,000 mouse E13.5 ovarian somatic cells in ULA 96-wells and co-cultured the mixture in RB27 supplemented with 100 ng/ml SCF, 50 ng/ml EGF and 10 μM ROCKi, for 2 days. After aggregation of both cell types, cellular clumps were cultured in an air-liquid interface system, as described before^42^. Briefly, cellular aggregates were place on 0,35% SeaKem LE Agarose (Lonza) stands, which were socked and surrounded by MEMα medium (Thermo Fisher Scientific) supplemented with 10% FBS (Thermo Fisher Scientific), 1% (vol/vol) PenStrep, 5 μM 2-mercaptoethanol and 150 μM Ascorbic acid (Sigma). Aggregates were cultured at 37 °C, in 5% CO_2_ and media was changed every 4 days.

#### Immunofluorescence staining of cryo-sections and cells

Human embryonic samples, embryoid bodies, and human hindgut organoid or mouse ovarian somatic cells co-culture aggregates were fixed in 4% (w/v) formaldehyde solution (Sigma) for 2 hours at 4 °C. Samples were washed with PBS and afterwards sequentially cryoprotected with 10% and 20% (w/v) sucrose (Sigma) at 4°C. Samples were then embedded in optimal cutting temperature compound (OCT) and subsequently snap-frozen in dry ice. Finally, frozen samples were sectioned at 8 μm thickness on Superfrost Plus Micro slides (Thermo Fisher Scientific) by a Leica 3050S cryostat. For cells cultivated on μ-Slide 8 well chamber (Thistle Scientific), we fixed these in 4% PFA for 10 min at 4°C, and subsequently washed twice with PBS. Immunofluorescence staining was performed as previously described^11,17^. Heat-mediated antigen retrieval in TE buffer (pH 8) at 95°C by a microwave oven for 40 minutes was performed before the incubation of primary antibodies against 5mC. Primary antibodies are listed in the key resources table. At the time of secondary antibody incubation samples were also counter stained with DAPI (Sigma). Imaging acquisition was accomplished with Leica TCS SP5 or SP8 confocal microscopes.

#### Cell sorting and analysis

Embryoid bodies, mouse ovarian somatic cell co-culture aggregates, and human gonadal ridges were dissociated with 0.25% trypsin/EDTA into single cells. Hindgut organoid co-cultures were sequentially dissociated with 0.25% trypsin/EDTA and 3 mg/ml collagenase (Sigma) into single cells. Human PGCLCs were either sorted based on the expression of NANOS3−tdTomato or also stained with Alexa-488 or -647 anti-TNAP antibody and Alexa-647 anti-PDPN. Human PGCs were stained with Alexa-488 anti-TNAP and ACP anti-CD117 antibodies. All conjugated antibodies are listed in the key resources table. Cell sorting was carried using a Sony SH800 Cell Sorter.

#### RNA-sequencing libraries preparation

Total RNA from 1,000−4,000 sorted cells was extracted using the PicoPure™ RNA Isolation Kit (Thermo Fisher Scientific), following providers’ instructions. RNA-seq libraries were prepared from 3−10 ng of total RNA using the NEBNext® Single Cell/Low Input RNA Library Prep Kit for Illumina® (New England BioLabs), following providers’ instructions. Libraries were quantified by q-PCR using the NEBNext Library Quant Kit Quick Protocol (New England BioLabs). Pooled libraries were subject to paired-end 50 or 100 bp sequencing on the NovaSeq 6000 sequencing system (Illumina). Every 48 indexed libraries were multiplexed to one lane of a S2 flowcell, resulting in > 40 million single-end reads per sample. RNA-sequencing libraries preparation was performed in three different batches.

### Quantification And Statistical Analysis

#### Immunostaining quantifications

After Immunofluorescence staining, cryo-sectioned samples were analysed under a fluorescence microscope. We counted NANOS3−tdTomato and NANOS3−tdTomato/DAZL double positive cells. Then, we calculated the percentage of NANOS3−tdTomato/DAZL double positive cells in the population of hPGCLCs (NANOS3−tdTomato positive cells), allowing comparison of different conditions. From all immunostaining quantifications, we counted more approximately 59,000 NANOS3−tdTomato positive cells and 680 NANOS3−tdTomato/DAZL double positive cells. Statistical details for each of these analyses can be found in the figure legends.

To quantify the average nuclear fluorescence intensity for 5mC and H3K9me2 staining, confocal stacks were maximum intensity Z-projected and converted to 8-bit signal depth. The channel containing DAPI-stained nuclear signal was segmented using a custom python script for Fiji that is available on request. In brief, the python script employs a Differences of Gaussians frequency bandpass of 0.8 to 1.6 μm and creates a Huang thresholded mask. The mask is further segmented with the watershed, and objects outside an 8−60 μm^2^ range were excluded. The mean intensity value was calculated for each nucleus. Nuclei expressing OCT4 and TFAP2C or OCT4 and NANOS3−tdTomato were defined as PGCLCs. The remaining nuclei were considered neighbouring somatic cells. The distribution of nuclear fluorescence intensity from both populations was plotted as boxplots. Quantification of the average nuclear fluorescence intensity for 5mC and H3K9me2 was based on confocal images of at least 3 independent embryoids for each condition. Statistical details for each of these analyses can be found in the figure legends.

#### Cell sorting and analysis

Cell sorting analyses were performed with the provided Sony Cell Sorter Software.

#### Bioinformatics analysis

The sequencing quality of RNA-seq libraries were checked by FastQC (v0.11.5). The adaptor sequences were removed by Flexbar (3.5.0) as specified by NEB (https://github.com/nebiolabs/nebnext-single-cell-rna-seq) with additional options (“--qtrim TAIL -- qtrim-format i1.8 -qt 20”) to remove low quality reads. The pre-processed reads were mapped to the human reference genome (UCSC GRCh38/hg38) using STAR (2.7.1a) (parameters: ‘--outFilterMismatchNoverLmax 0.05 --outFilterMultimapNmax 50 --outMultimapperOrder Random’) guided by the Gencode Human Release 30 comprehensive gene annotation. Raw read counts per gene were extracted by the featureCounts function of the Subread package (1.6.2) using the default parameters. Only ‘protein_coding’ and ‘lincRNA’ genes were considered for subsequent analysis. Normalized read counts [log2(normalized counts +1)] and differentially expressed genes (absolute(log2(fold change)) > 2 and adjusted p-value < 0.05) were obtained using DEseq2 (1.26.0) in R (3.6.2) /Bioconductor (3.10.1). PCA was performed using the R prcomp function..

## Supplementary Material

Supplementary Material

## Data Availability

RNA sequencing data have been deposited at Gene Expression Omnibus database, and is available as of the date of publication under accession number: GSE203156. This paper does not report original code. Any additional information required to reanalyse the data reported in this paper is available from the lead contact upon request.
