# Supplementary material for: Specification of human germ cell fate with enhanced progression capability supported by hindgut organoids
Source: Cell Rep. Author manuscript; Available in PMC 2025 Sep 8. (PMC7618081; doi:10.1016/j.celrep.2022.111907)
Supplement: Supplementary Material [file EMS208342-supplement-Supplementary_Material.pdf]

Figure S1: supports figure 1

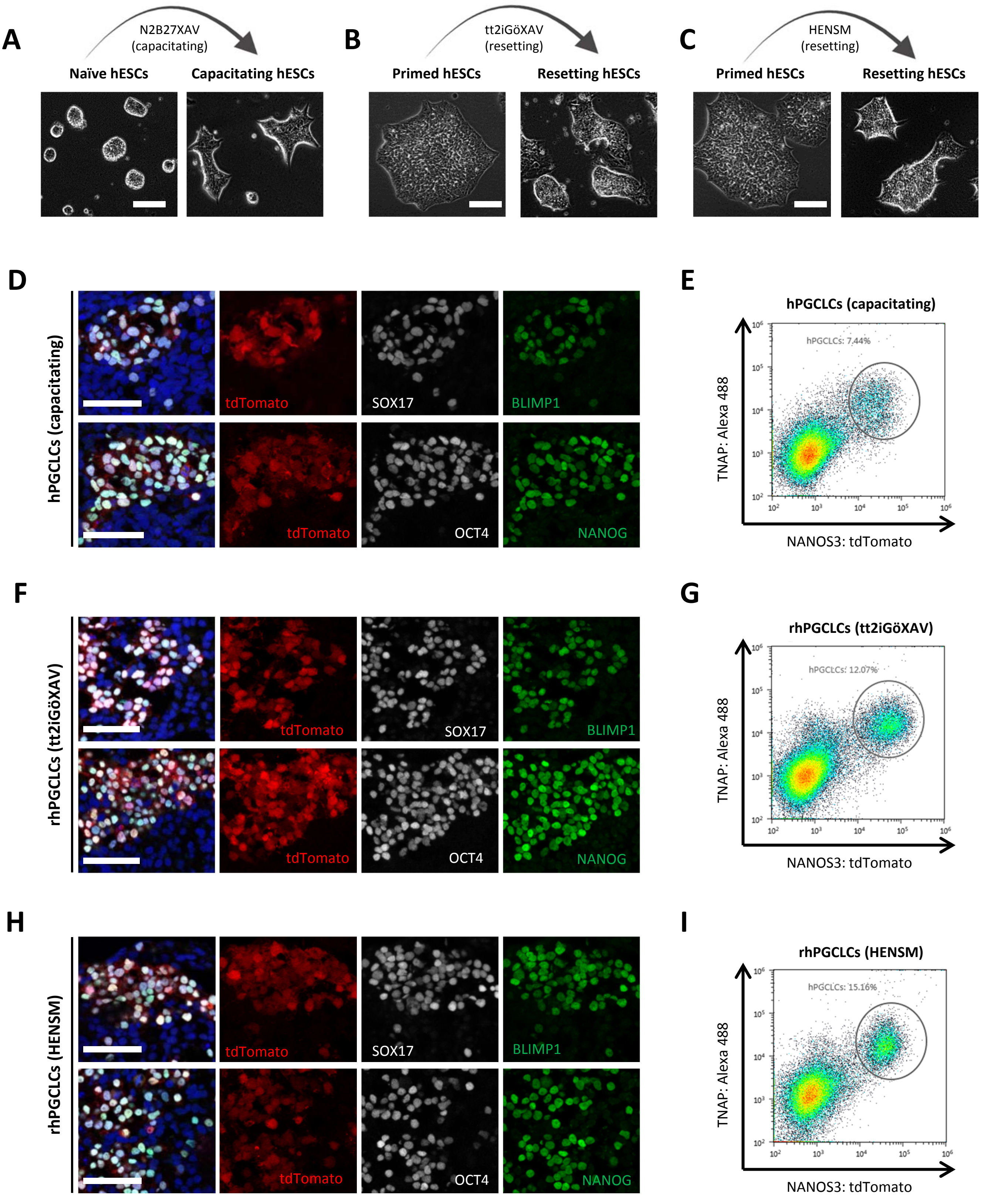

Figure S1. Characterization of capacitating and resetting precursors and hPGCLCs, Related to Figure 1
(A−C) Bright-field images for the conversion of (A) naïve hESCs (P10) to capacitating hESCs (day 3), (B) primed hESCs to resetting hESCs (tt2iGöXAV, P3), and (C) primed hESCs to resetting hESCs (HENSM, P2). Scale bar, 100 um. Arrows point the direction of conversions. (D, F, and H) Immunofluorescence of OCT4, SOX17, NANOG and BLIMP1 on sections from day 4 embryoid bodies containing hPGCLCs expressing NANOS3−tdTomato specified from W15 (D) capacitating, (F) resetting (tt2iGöXAV), and (H) resetting (HENSM) hESCs. DAPI nuclear counterstain showed in blue. Scale bar, 50 um. (E, G, and I) Flow cytometry analysis plots showing the percentage of hPGCLCs co-expressing NANOS3−tdTomato and TNAP in day 4 embryoid bodies generated from W15 (E) capacitating, (G) resetting (tt2iGöXAV), and (I) resetting (HENSM) hESCs.

Figure S2: supports figure 1

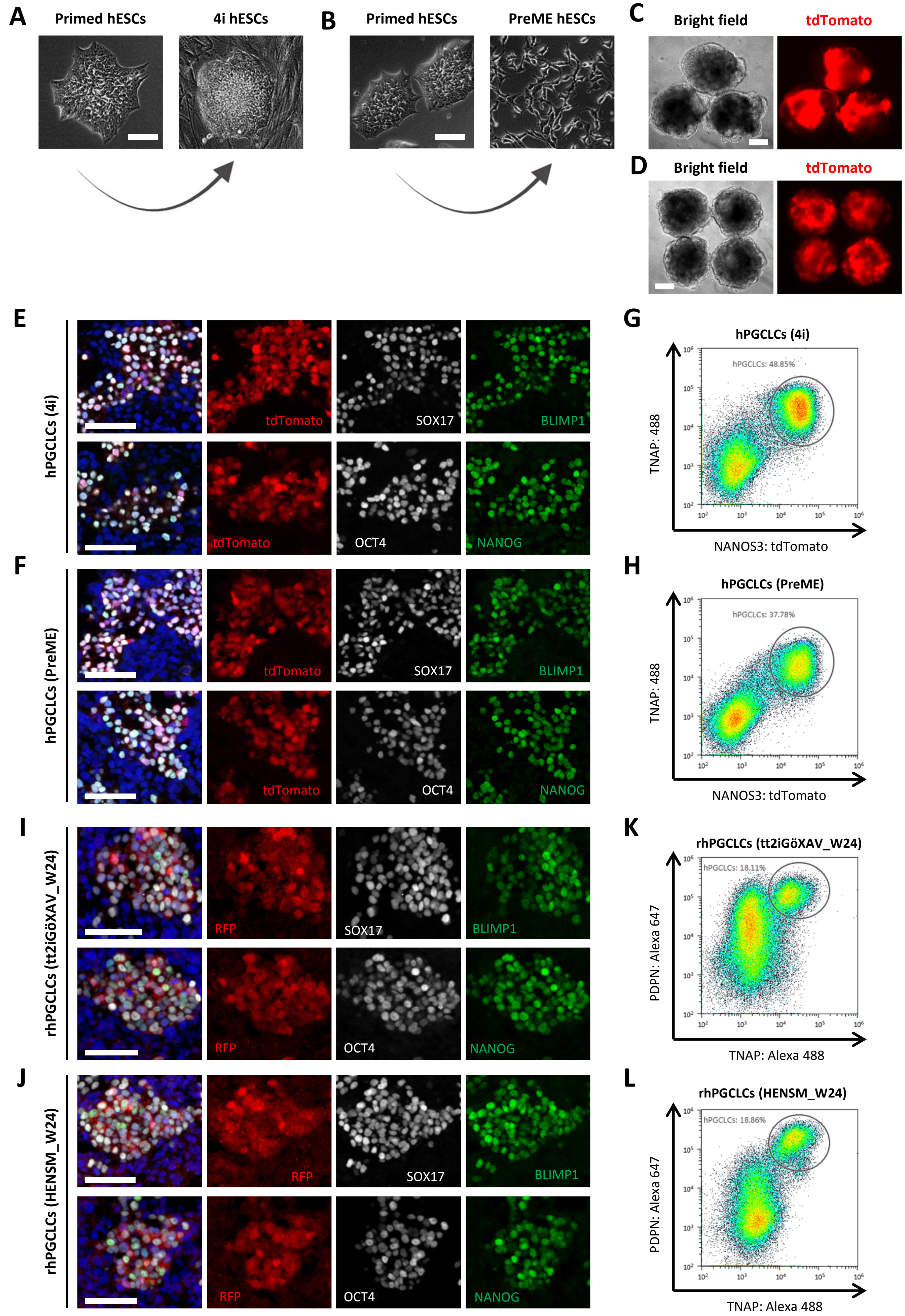

Figure S2. Characterization of resetting and peri-gastrulation precursors and hPGCLCs, Related to Figure 1 (A and B) Bright-field images for the conversion of (A) primed hESCs to 4i hESCs (P2), and (B) primed hESCs to PreME hESCs (12 hours). Scale bar, 100 um. Arrows point the direction of conversions. (C and D) Day 4 embryoid bodies generated from (C) 4i hESCs, and (D) PreME hESCs. Scale bar, 200 um. (E and F) Immunofluorescence of OCT4, SOX17, NANOG and BLIMP1 on sections from day 4 embryoid bodies containing hPGCLCs expressing NANOS3−tdTomato specified from W15 (E) 4i and (F) PreME hESCs. DAPI nuclear counterstain showed in blue. Scale bar, 50 um. (G and H) Flow cytometry analysis plots showing the percentage of hPGCLCs co-expressing NANOS3−tdTomato and TNAP in day 4 embryoid bodies generated from W15 (G) 4i and (H) PreME hESCs. (I and J) Immunofluorescence of OCT4, SOX17, NANOG, BLIMP1, and Red Fluorescent Protein (RFP) on sections from day 4 embryoid bodies containing hPGCLCs expressing NANOS3−mCherry specified from W24 (I) tt2iGöXAV (J) HENSM resetting hESCs. DAPI nuclear counterstain showed in blue. Scale bar, 50 um. (K and L) Flow cytometry analysis plots showing the percentage of hPGCLCs co-expressing PDPN and TNAP in day 4 embryoid bodies generated from W24 (K) tt2iGöXAV (L) HENSM resetting hESCs.

Figure S3: supports figure 1, 4 , and 5

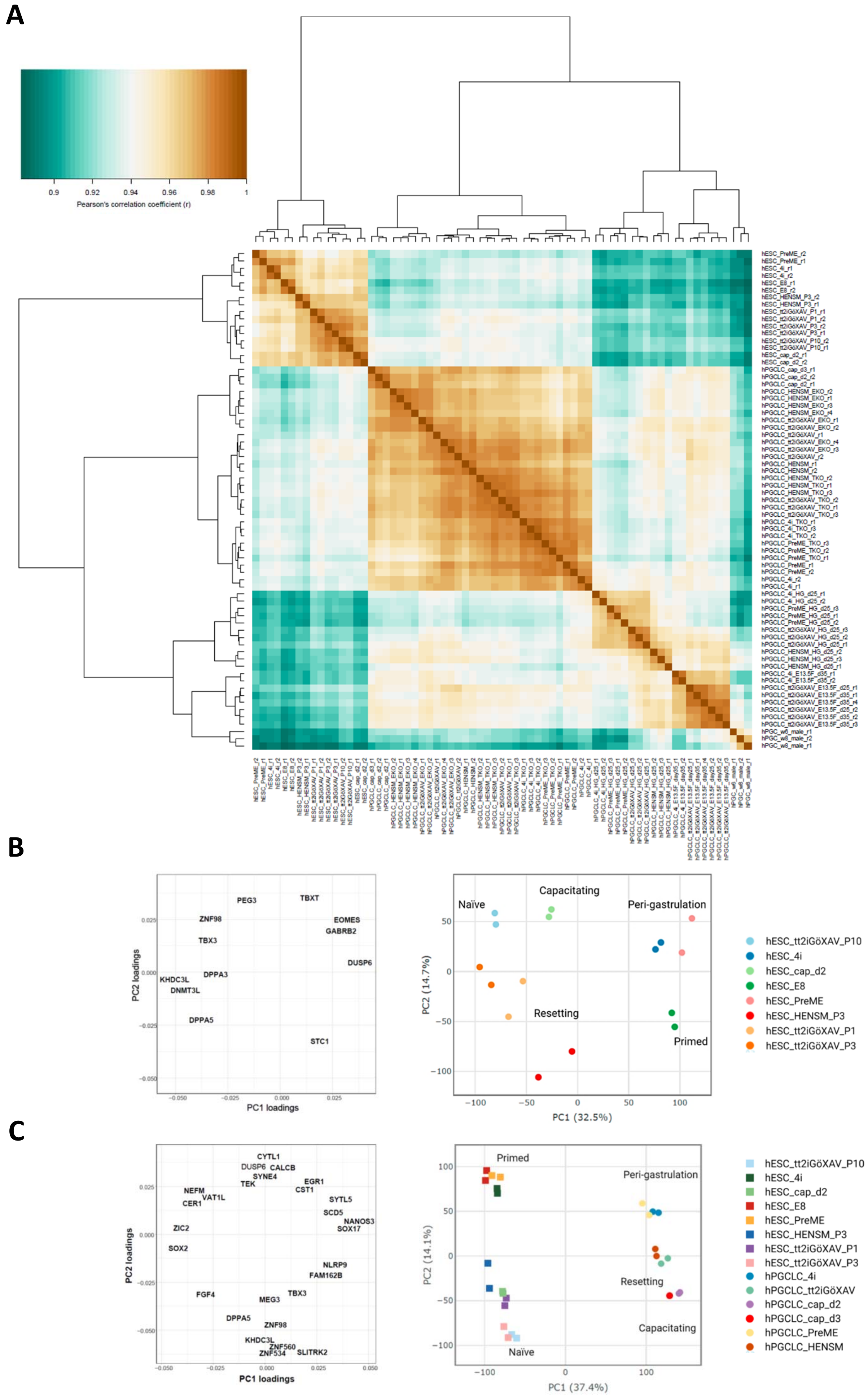

Figure S3. Clustering analysis of precursors, hPGCLCs, and hPGCs gene expression profiles, Related to Figure 1, 4, and 5 (A) Unsupervised hierarchical clustering analysis; heat map of pairwise Pearson correlation coefficients between the transcriptomic profiles of each of the precursors, hPGCLCs, co-cultured hPGCLCs, and hPGCs analysed. For precursors, passage (P) after resetting conversion and days (d) of capacitation (cap) are indicated. For hPGCLCs, EOMES (EKO) and TBXT (TKO) knockout backgrounds are indicated. Also, days (d) of capacitation (cap) for the respective precursors are shown. All hPGCLCs were analysed 4 days after induction. For co-cultured hPGCLCs, the co-culture period in days (d) and conditions [either hindgut organoid (HG) or mouse E13.5 ovarian somatic cell (E13.5F) co-cultures] are indicated. For hPGCs, developmental embryonic weeks (w) are specified. (B) Two-dimensional PCA plot (PC2 against PC1) and gene loading plot for hESCs and hPGCLC precursors from peri-gastrulation (4i and PreME), resetting tt2iGöXAV, resetting HENSM, and capacitating (cap) conditions. Passage after conversions (P) or days of capacitation (d) are indicated. (C) Two-dimensional PCA plot (PC2 against PC1) and gene loading plot for hESCs, hPGCLC precursors, and day 4 hPGCLCs from peri-gastrulation (4i and PreME), resetting tt2iGöXAV, resetting HENSM, and capacitating (cap) conditions. Passage after conversions (P) or days of capacitation (d) are indicated.

Figure S4: supports figure 1, 4 , and 5

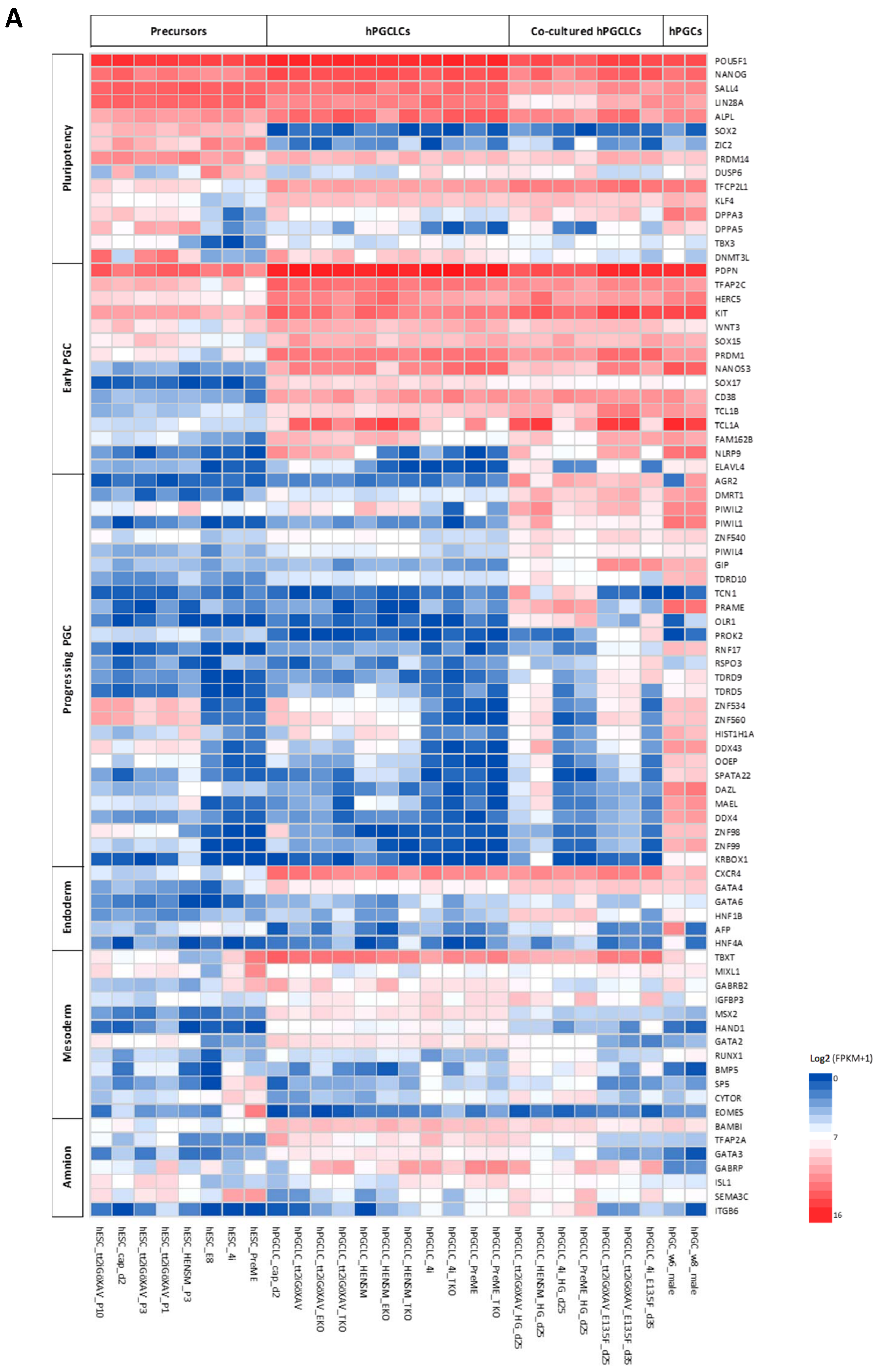

Figure S4. Transcriptomic profiles of precursors, hPGCLCs, and hPGCs, Related to Figure 1, 4, and 5

(A) Heat map displaying the mean expression of representative genes for human pluripotency, early hPGCs, progressing hPGCs, endoderm, mesoderm, and amnion in each of the precursors, hPGCLCs, co-cultured hPGCLCs, and hPGCs analysed. For precursors, passage (P) after resetting conversion and days (d) of capacitation (cap) are indicated. For hPGCLCs, EOMES (EKO) and TBXT (TKO) knockout backgrounds are indicated. Also, days (d) of capacitation (cap) for the respective precursors are shown. All hPGCLCs were analysed 4 days after induction. For co-cultured hPGCLCs, the co-culture period in days (d) and conditions [either hindgut organoid (HG) or mouse E13.5 ovarian somatic cell (E13.5F) co-cultures] are indicated. For hPGCs, developmental embryonic weeks (w) are specified.

Figure S5: supports figure 1

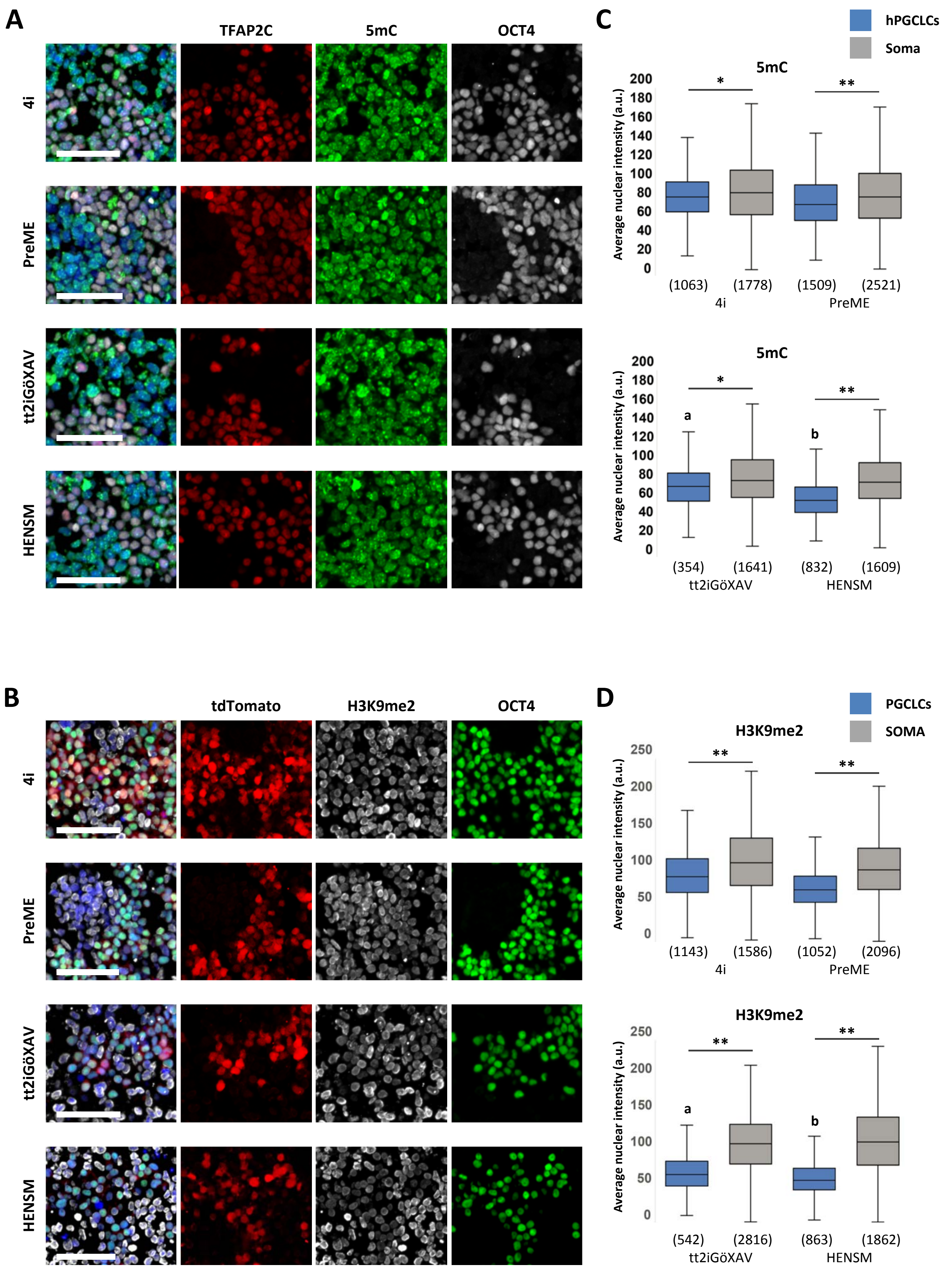

Figure S5. Epigenetic profile of resetting and peri-gastrulation hPGCLCs, Related to Figure 1, (A and B) Immunofluorescence of (A) TFAP2C, 5mC, and OCT4 and (B) H3K9me2 and OCT4 on sections from day 4 embryoid bodies containing hPGCLCs specified from W15 resetting (tt2iGöXAV or HENSM) and peri-gastrulation (4i or PreME) hESCs. DAPI nuclear counterstain showed in blue. Scale bar, 50 um. (C and D) Quantification of nuclear fluorescence intensity (arbitrary unit; a.u.) of (C) 5mC and (D) H3K9me2 on sections from day 4 embryoid bodies containing hPGCLCs and neighbouring somatic cells specified from W15 resetting (tt2iGöXAV or HENSM) and peri-gastrulation (4i or PreME) precursors. Boxplots show the distribution of data [median (midle line), interquartile range (boxes), minimum and maximum (whiskers)]. Numbers of cells analysed shown between brackets. At least n=3 measurements were taken from independent experiments and for each condition. T-test between hPGCLCs and somatic cells (SOMA) from the same condition: *p-value <0.01; **p-value <0.001. One-way ANOVA among the four different hPGCLC groups for 5mC (p-value =3.72E-40) and H3K9me2 (p-value =1.31E-117) followed by T-test corrected for multiple comparisons: [a] p-value <0.05 (compared with 4i); [b] p-value <0.001 (compared with 4i or PreME).

Figure S6 (supports Figure 2)

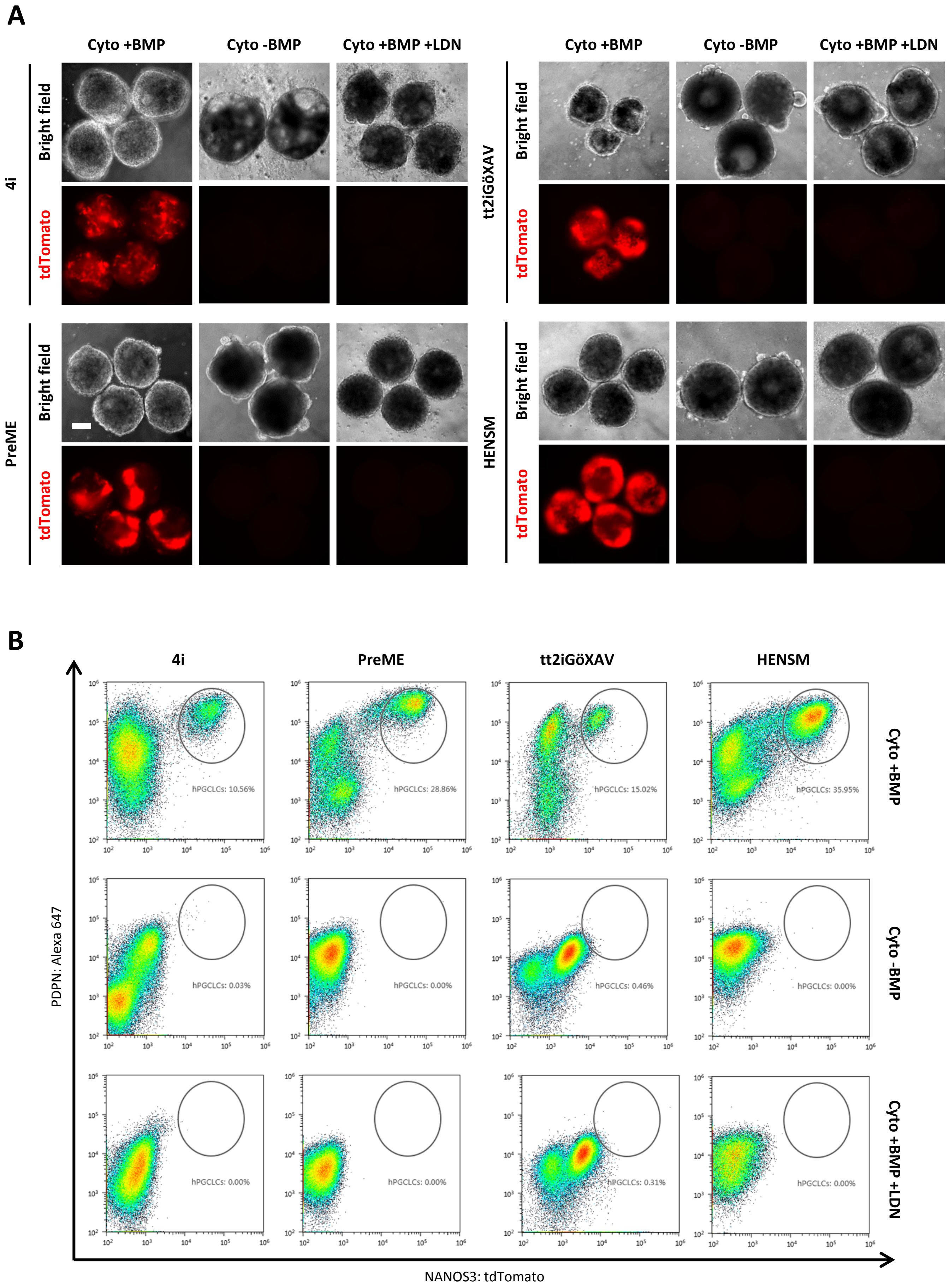

Figure S6. Dependency of BMP for hPGCLC specification from peri-gastrulation and resetting precursors, Related to Figure 2 (A) Day 4 embryoid bodies generated from peri-gastrulation (4i and PreME) and resetting (tt2iGöXAV and HENSM) NANOS3−tdTomato precursors, under three different experimental conditions (Cyto+BMP, Cyto-BMP, and Cyto+BMP+LDN). hPGCCs in the embryoid bodies expressed NANOS3−tdTomato. Scale bar, 200 um. (B) Flow cytometry analysis plots showing the percentage of hPGCLCs co-expressing NANOS3−tdTomato and PDPN in day 4 embryoid bodies generated from peri-gastrulation (4i and PreME) and resetting (tt2iGöXAV and HENSM) NANOS3−tdTomato precursors, under three different experimental conditions (Cyto+BMP, Cyto-BMP, and Cyto+BMP+LDN).

Figure S7 (supports figure 2)

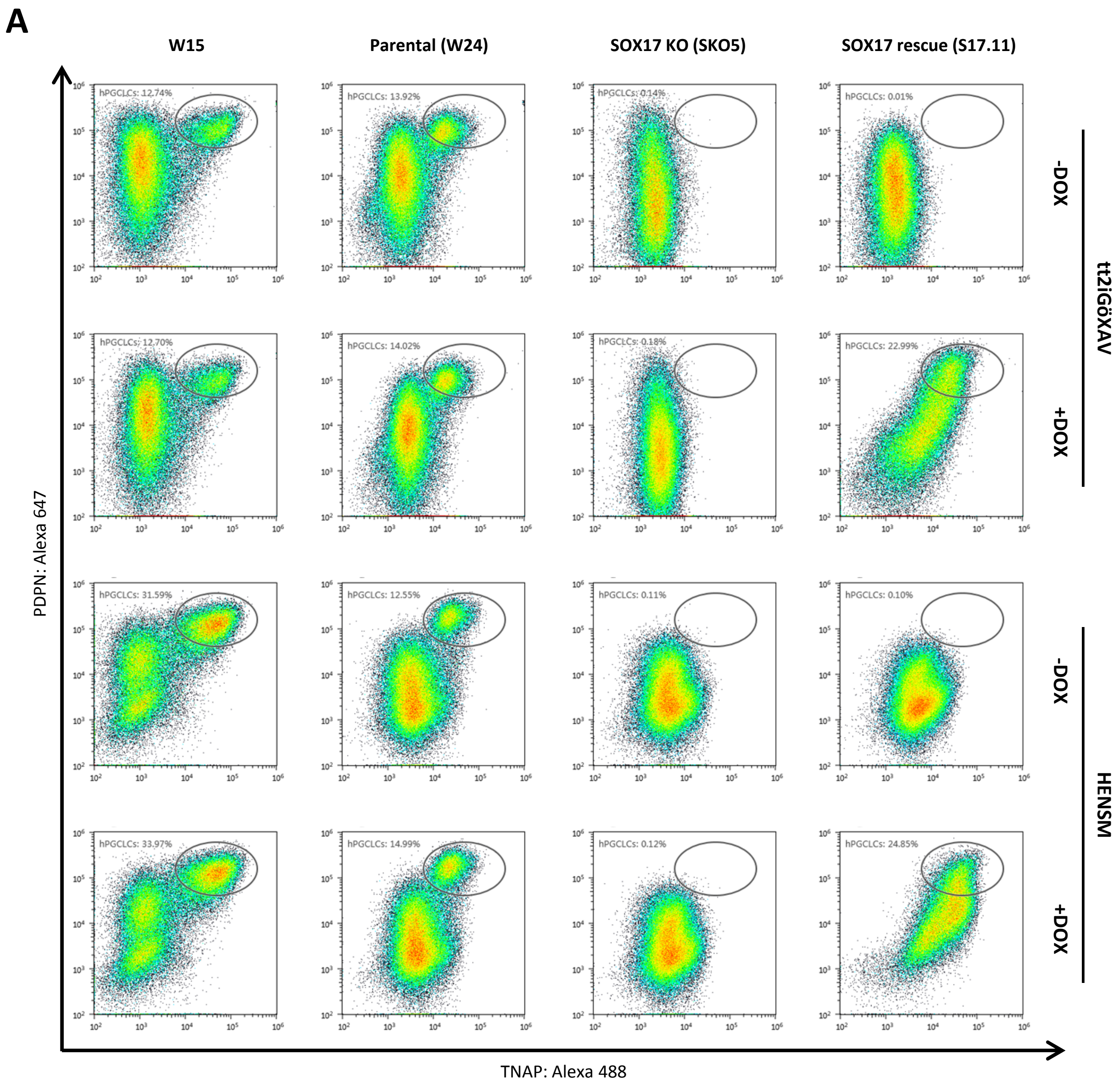

Figure S7. Dependency of SOX17 for hPGCLC specification from resetting precursors, Related to Figure 2 (A) Flow cytometry analysis plots showing the percentage of hPGCLCs co-expressing TNAP and PDPN in day 4 embryoid bodies generated from resetting precursors (tt2iGöXAV and HENSM) in four different genetic backgrounds (W15, parental W24, SOX17 knockout (SKO5), and SOX17 rescue (S17.11) lines), under the absence or presence of DOX.

Figure S8 (supports figure 3)

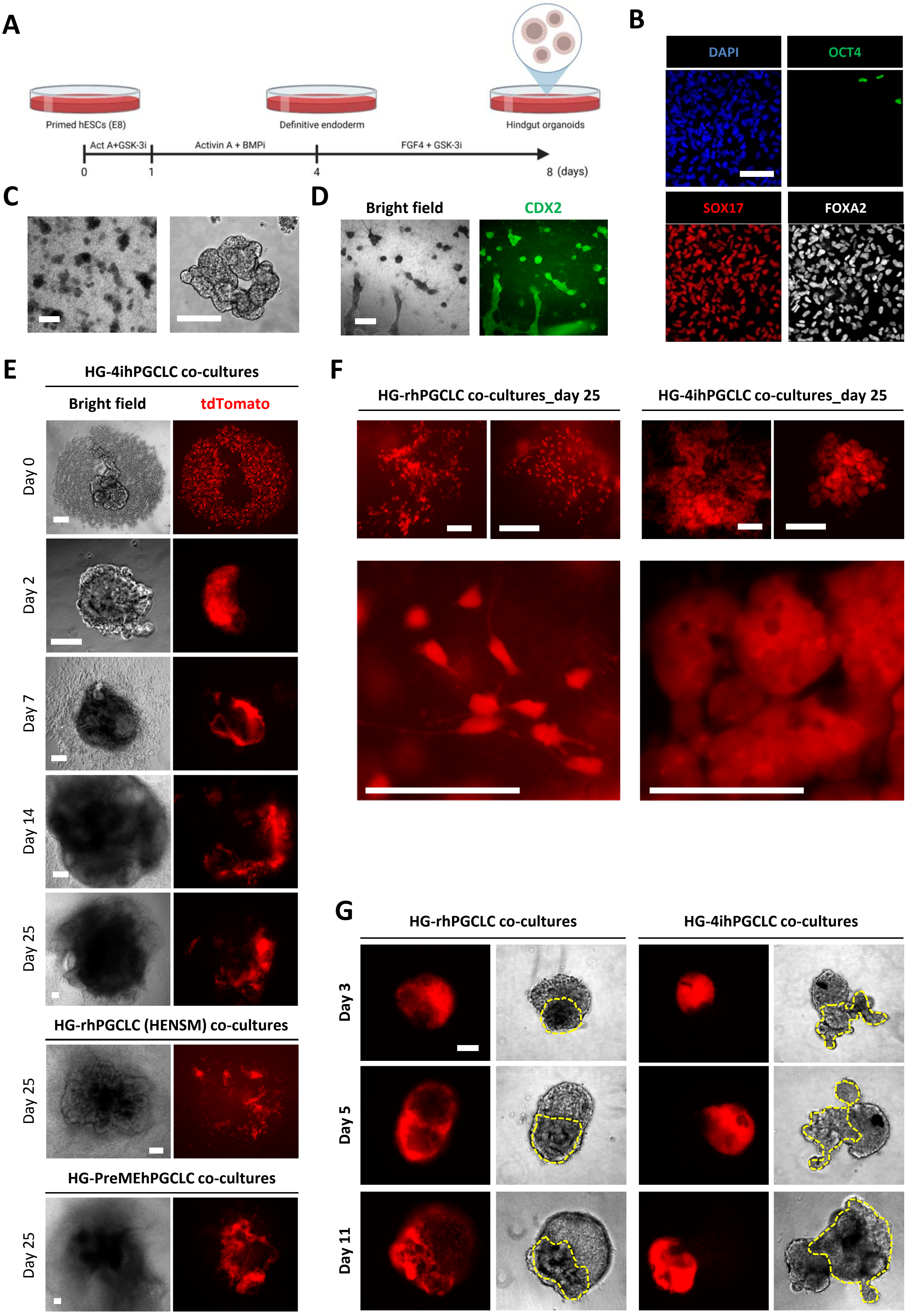

Figure S8. Human hindgut organoid specification and hPGCLC distribution in the co-cultures with human hindgut organoids, Related to Figure 3 (A) Schematic diagram for human hindgut organoid induction protocol. (B) Immunofluorescence of OCT4, SOX17, and FOXA2 on day 4 human definitive endoderm. DAPI nuclear counterstain showed in blue. Scale bar, 100 um. (C) Bright-field imaging for day 8 human hindgut organoids. Scale bar, 200 um. (D) Immunofluorescence of CDX2 on day 8 human hindgut organoids. Scale bar, 200 um. (E) Co-cultures of human hindgut organoids (HG) and NANOS3−tdTomato peri-gastrulation (4i) hPGCLCs over 25 days, resetting (HENSM) for 25 days, and peri-gastrulation (PreME) hPGCLCs for 25 days. Scale bar, 200 um. (F) Fluorescence imaging for resetting (tt2iGöXAV, left) and peri-gastrulation (4i, right) hPGCLCs expressing NANOS3−tdTomato in day 25 human hindgut organoid (HG) co-cultures. Scale bar, 200 um. (G) Co-culture of human hindgut organoids (HG) with NANOS3−tdTomato resetting (tt2iGöXAV, left) and peri-gastrulation (4i, right) hPGCLCs over 11 days, in ultra-low attachment wells. Scale bar, 200 um.

Figure S9 (supports figure 3)

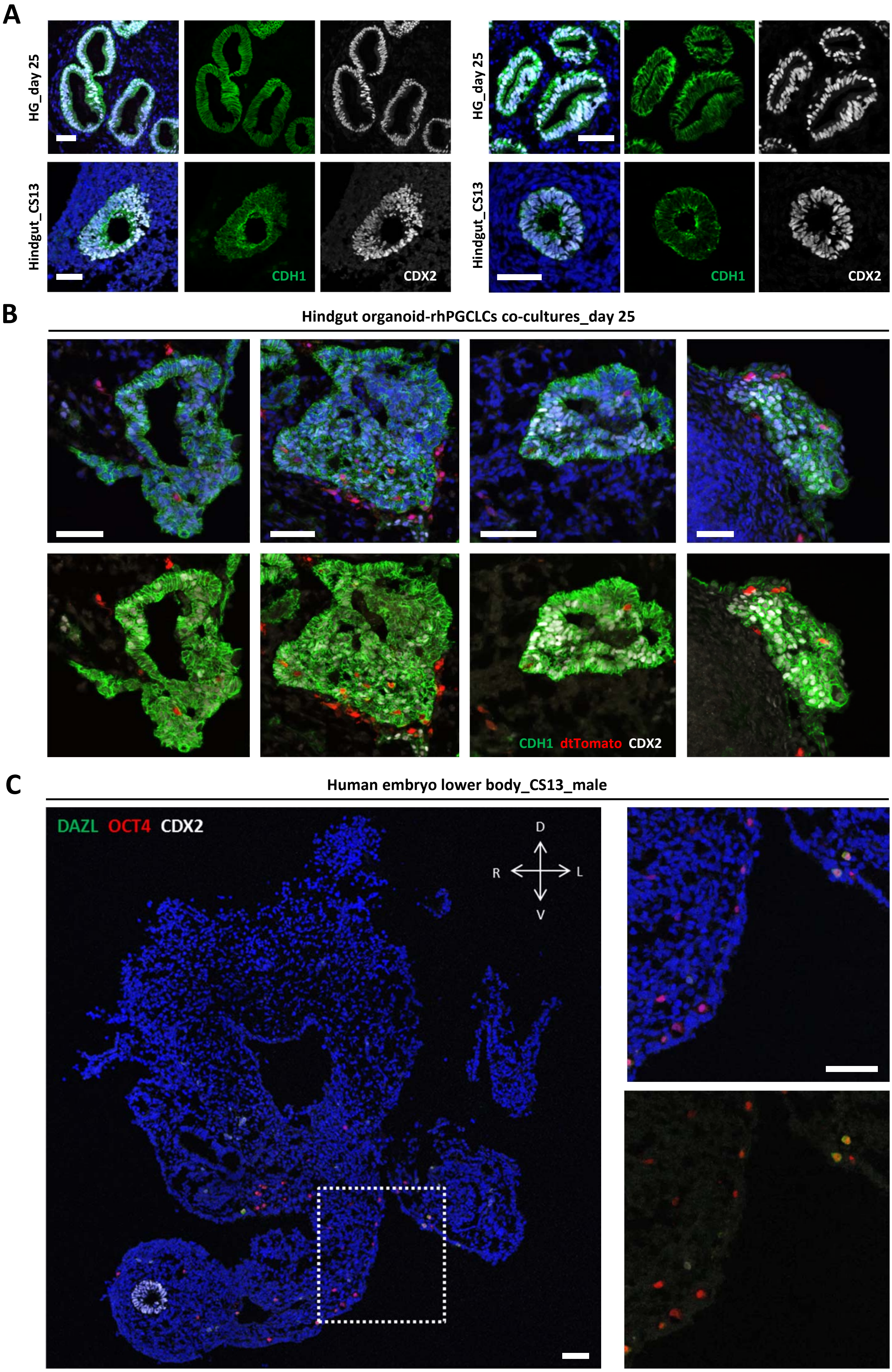

Figure S9. Human hindgut and hindgut organoid co-culture characterization, Related to Figure 3 (A) Immunofluorescence of CDH1 and CDX2 on sections of day 25 human hindgut organoid (HG) cultures (top panels) and on sections of a CS13 human hindgut (bottom panels). DAPI nuclear counterstain showed in blue. Scale bar, 50 um. (B) Immunofluorescence of CDH1 and CDX2 on section of a day 25 human hindgut organoid co-culture containing resetting (tt2iGöXAV) hPGCLCs expressing NANOS3−tdTomato. DAPI nuclear counterstain showed in blue. Scale bar, 50 um. (C) Immunofluorescence of OCT4, CDX2, and DAZL on sections of the lower body of a CS13 human embryo. Ventral (V), dorsal (D), left (L), and right (D). DAPI nuclear counterstain showed in blue. Scale bar, 50 um.

Figure S10 (supports figure3)

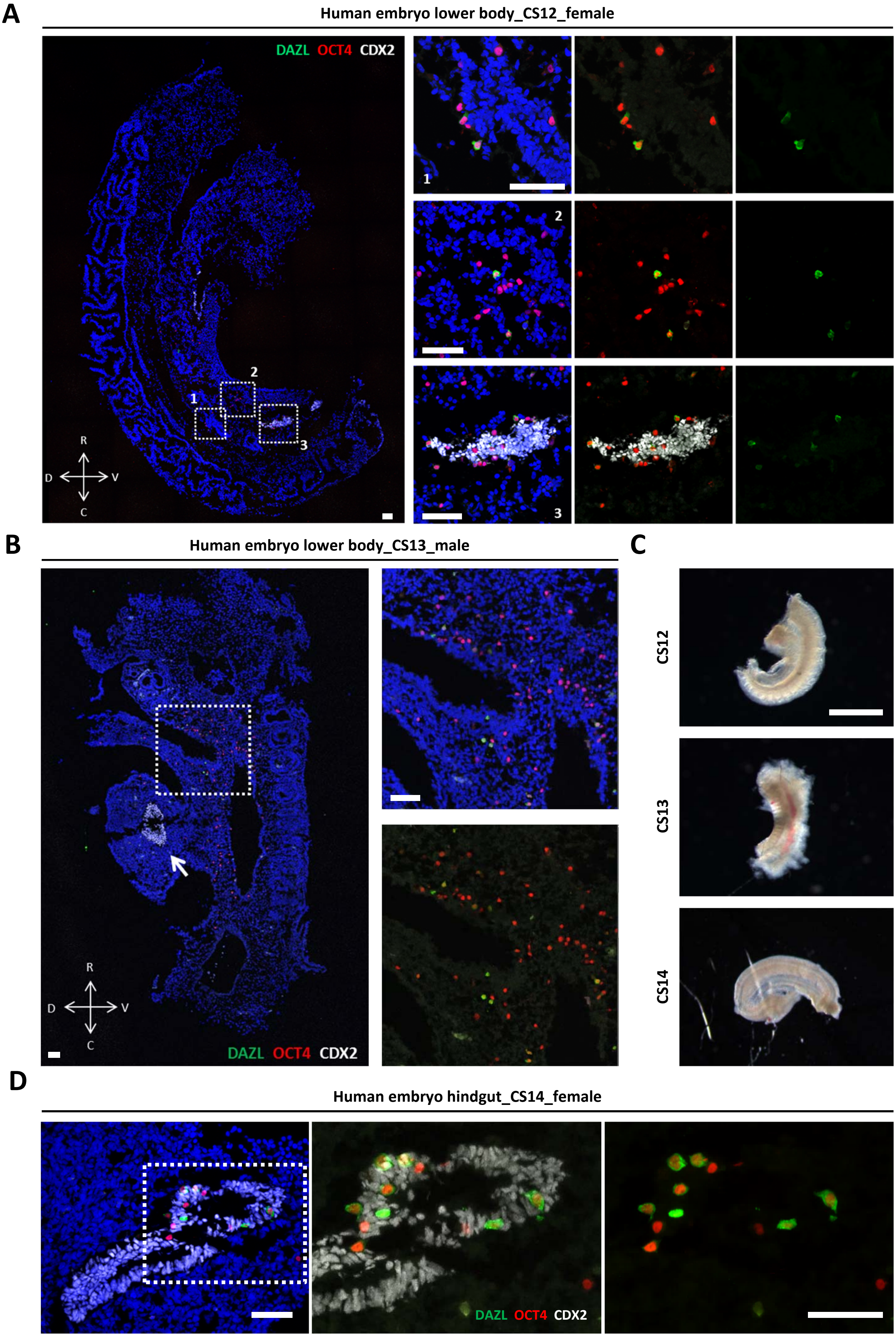

Figure S10. DAZL expression in hPGCs form CS12−CS13 human embryos, Related to Figure 3 (A and B) Immunofluorescence of OCT4, CDX2, and DAZL on sections of the lower body of (A) CS12 and (B) CS13 human embryos. Ventral (V), dorsal (D), left (L), and right (D). Scale bar, 50 um. (C) Bright-field imaging for CS12 (top) and CS13 (middle, dissected) lower body of human embryos. Bright-field imaging for hindgut and dorsal mesentery tissues (bottom) from a CS14 human embryo. Scale bar, 1000 um. (D) Immunofluorescence of OCT4, CDX2, and DAZL on a section of hindgut and dorsal mesentery tissues from a CS14 human embryo. DAPI nuclear counterstain showed in blue. Scale bar, 50 um.

Figure S11 (Supports figure 3 and 4)

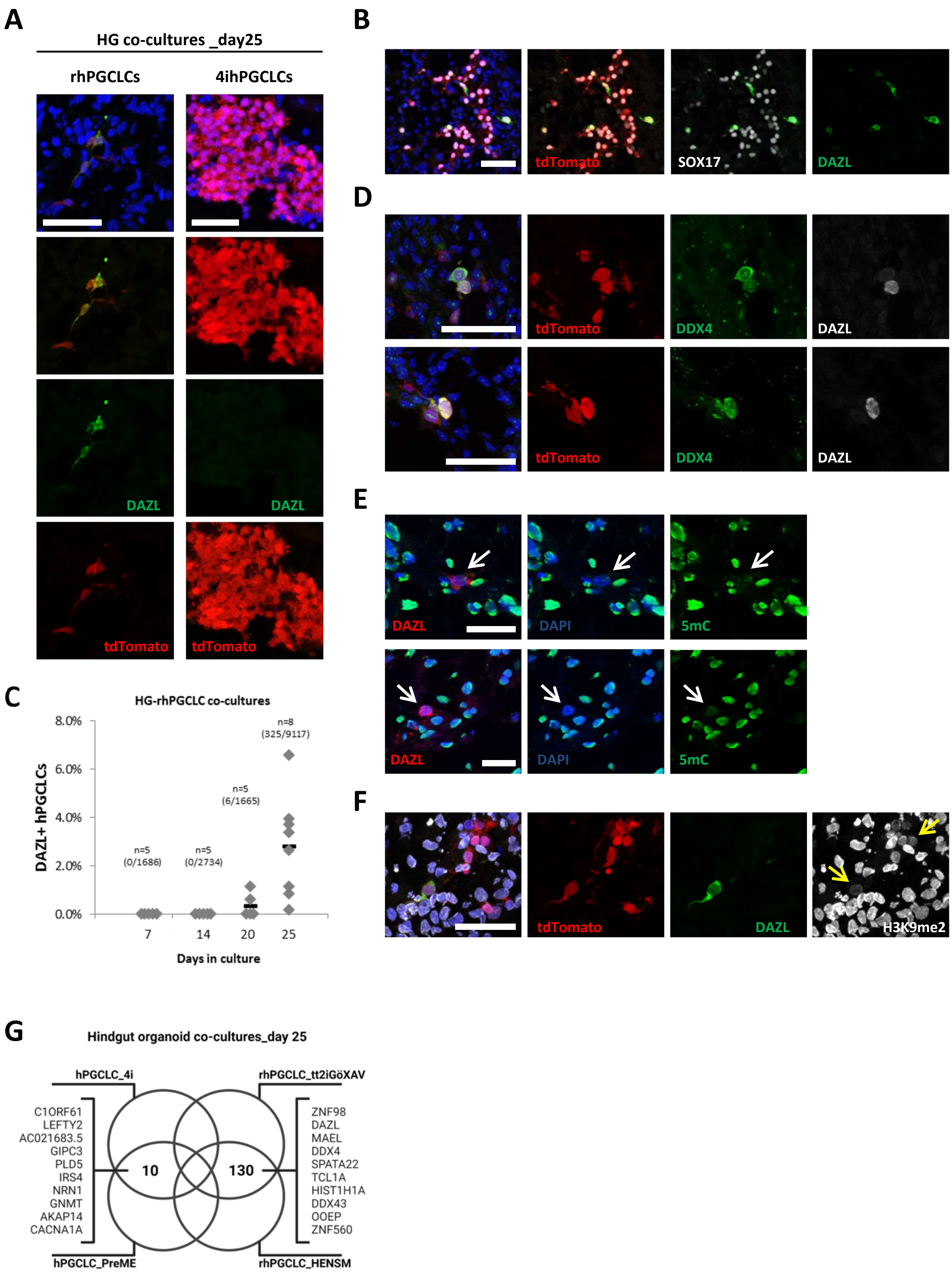

Figure S11. Characterization of progressed resetting hPGCLCs co-cultured with human hindgut organoids, Related to Figure 3 and 4 (A) Immunofluorescence of DAZL on sections of day 25 human hindgut organoid (HG) co-cultures containing resetting (tt2iGöXAV, left) and peri-gastrulation (4i, right) hPGCLCs expressing NANOS3−tdTomato. DAPI nuclear counterstain showed in blue. Scale bar, 50 um. (B) Immunofluorescence of SOX17 and DAZL on a section of a day 25 human hindgut organoid co-culture containing resetting (tt2iGöXAV) hPGCLCs expressing NANOS3−tdTomato. DAPI nuclear counterstain showed in blue. Scale bar, 50 um. (C) Percentage of DAZL positive resetting (tt2iGöXAV) hPGCLCs out of NANOS3-tdTomato positive hPGCLCs over 25 days of co-culture period with hindgut organoids (HG). Horizontal bars represent the mean percentage for each time point. At least n=5 measurements were taken from independent experiments for each time point. Total number of cells counted (double DAZL and tdTomato positive/ tdTomato positive cells) per time point shown between brackets. At least n=5 measurements were taken from independent experiments and for each time point. (D) Immunofluorescence of DAZL and DDX4 on sections of day 25 human hindgut organoid co-cultures containing resetting (tt2iGöXAV) hPGCLCs expressing NANOS3−tdTomato. DAPI nuclear counterstain showed in blue. Scale bar, 50 um. (E) Immunofluorescence of DAZL and 5mC on sections of day 25 human hindgut organoid co-cultures containing resetting (tt2iGöXAV) hPGCLCs. DAPI nuclear counterstain showed in blue. Arrows point to hPGCLCs with low 5mC expression. Scale bar, 25 um. (F) Immunofluorescence of DAZL and H3K9me2 on a section of a day 25 human hindgut organoid co-culture containing resetting (tt2iGöXAV) hPGCLCs expressing NANOS3−tdTomato. DAPI nuclear counterstain showed in blue. Arrows point to hPGCLCs with low H3K9me2 expression. Scale bar, 50 um. (G) Venn diagram showing differentially expressed genes ($log_2FC > 2$ and adjusted p-value < 0.05) commonly upregulated in peri-gastrulation hPGCLCs (4i and PreME) versus rhPGCLCs (tt2iGöXAV and HENSM) co-cultured with human hindgut organoids for 25 days, and vice versa.

Figure S12 (supports figure 4)

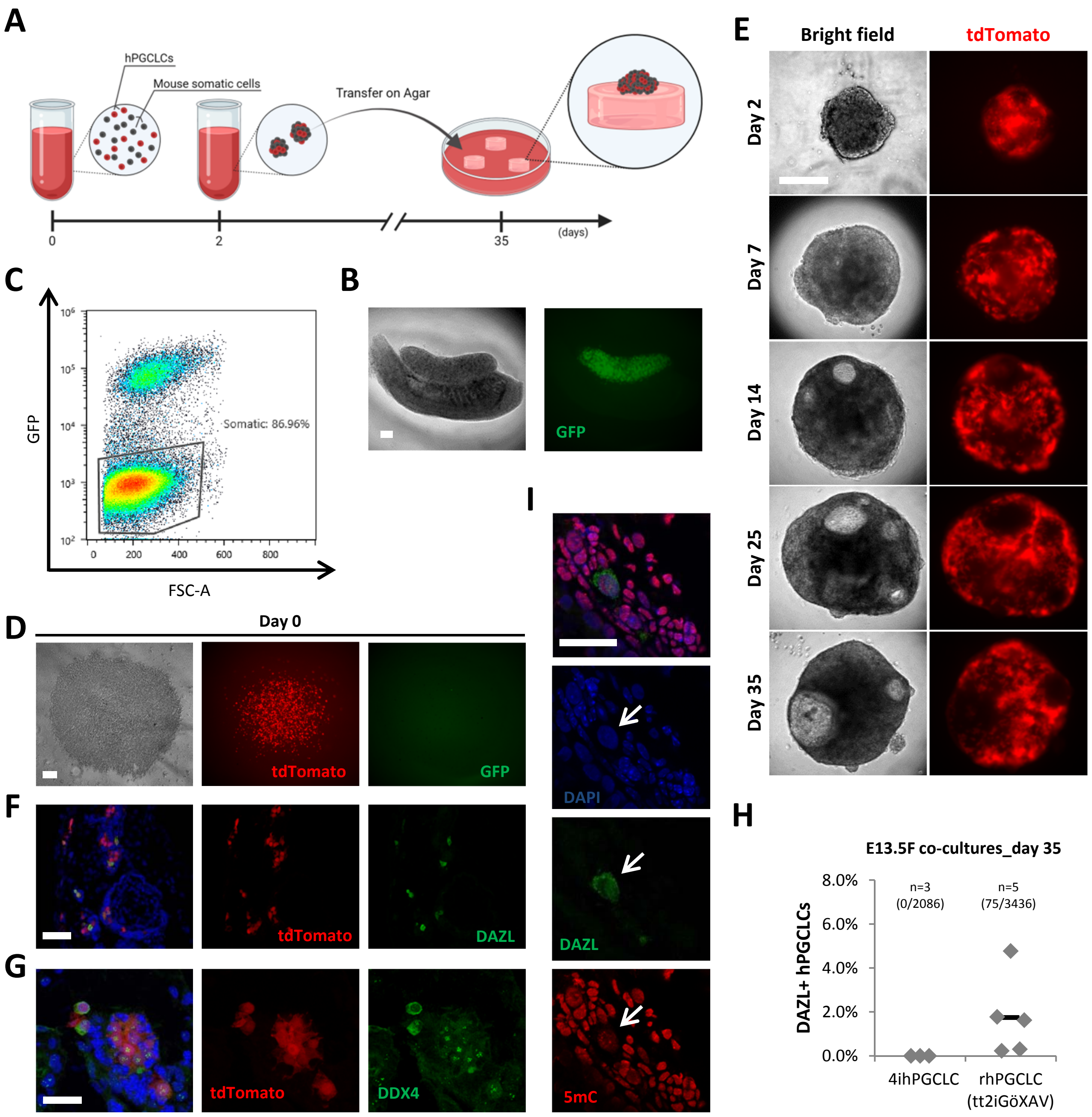

Figure S12. Progression of resetting hPGCLCs supported by mouse ovarian somatic cells, Related to Figure 4 (A) Schematic diagram for the co-culture strategy of hPGCLCs with mouse E13.5 ovarian somatic cells. (B) Mouse E13.5 mesonephros and ovary containing GFP-positive mPGCs. Scale bar, 200 um. (C) FACS plot for gating strategy to sort the GFP-negative mouse E13.5 ovarian somatic cells. (D) Co-culture of GFP-negative mouse E13.5 ovarian somatic cells with resetting (tt2iGöXAV) NANOS3−tdTomato hPGCLCs , in a ultra-low attachment well, at day 0. Scale bar, 200 um. (E) Co-culture GFP-negative mouse E13.5 ovarian somatic cells with resetting (tt2iGöXAV) NANOS3−tdTomato hPGCLCs over 35 days. Scale bar, 200 um. (F and G) Immunofluorescence of DAZL (F) and DDX4 (G) on sections of day 35 mouse E13.5 ovarian somatic cell co-cultures containing resetting (tt2iGöXAV) hPGCLCs expressing NANOS3−tdTomato. DAPI nuclear counterstain showed in blue. Scale bars, (F) 50 and (G) 25 um. (H) Percentage of DAZL positive peri-gastrulation (4i) or resetting (tt2iGöXAV) hPGCLCs out of NANOS3-tdTomato positive hPGCLCs in day 35 mouse E13.5 ovarian somatic cell (E13.5F) co-cultures. Horizontal bars represent the mean percentage for each condition. At least n=3 measurements were taken from independent experiments and for each condition. Total number of cells counted (double DAZL and tdTomato positive/ tdTomato positive cells) per condition shown between brackets. (I) Immunofluorescence of DAZL and 5mC on sections of day 35 mouse E13.5 ovarian somatic cell co-cultures containing resetting (tt2iGöXAV) hPGCLCs. DAPI nuclear counterstain showed in blue. Scale bar, 25 um.

Figure S13 (supports figure 5)

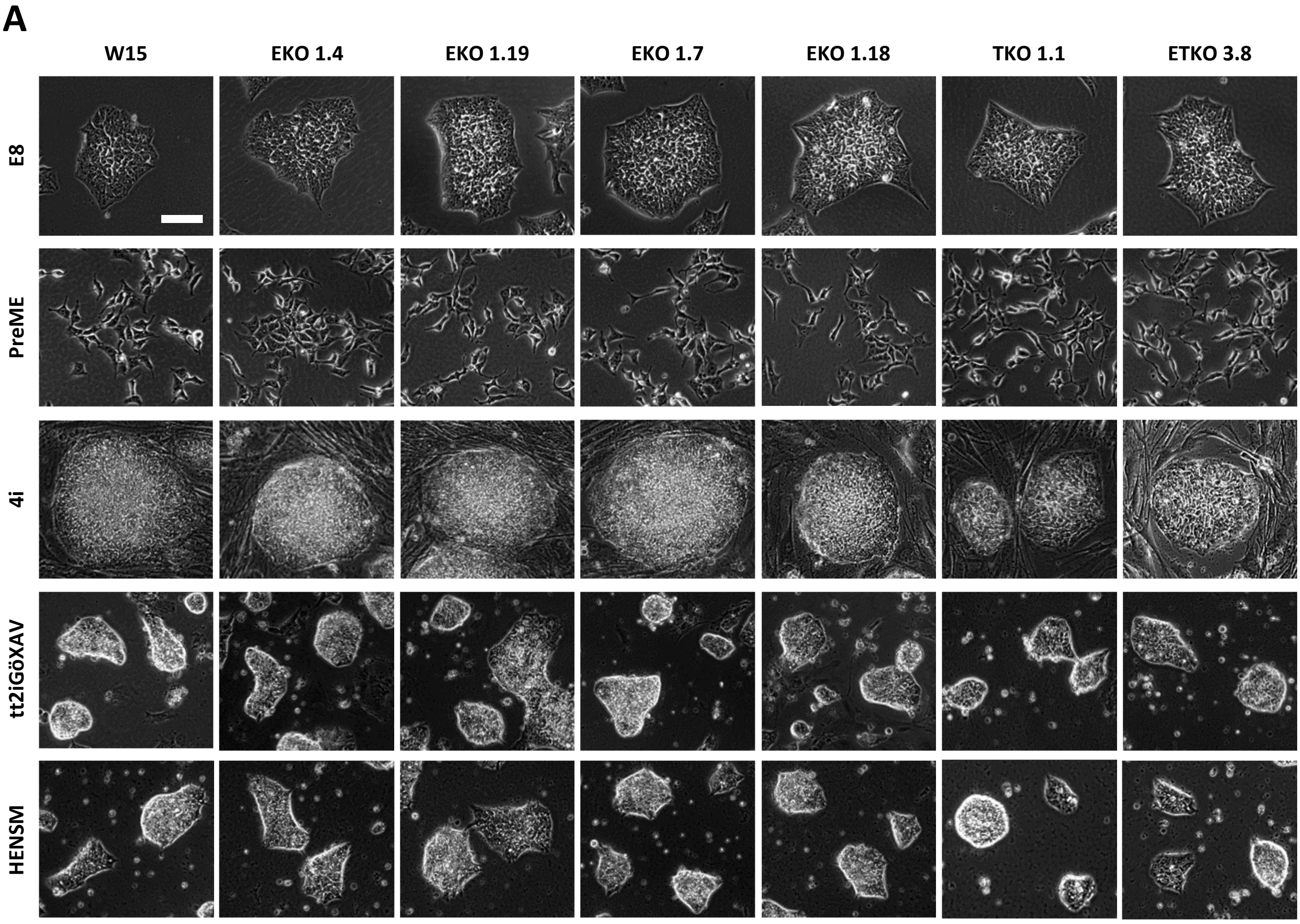

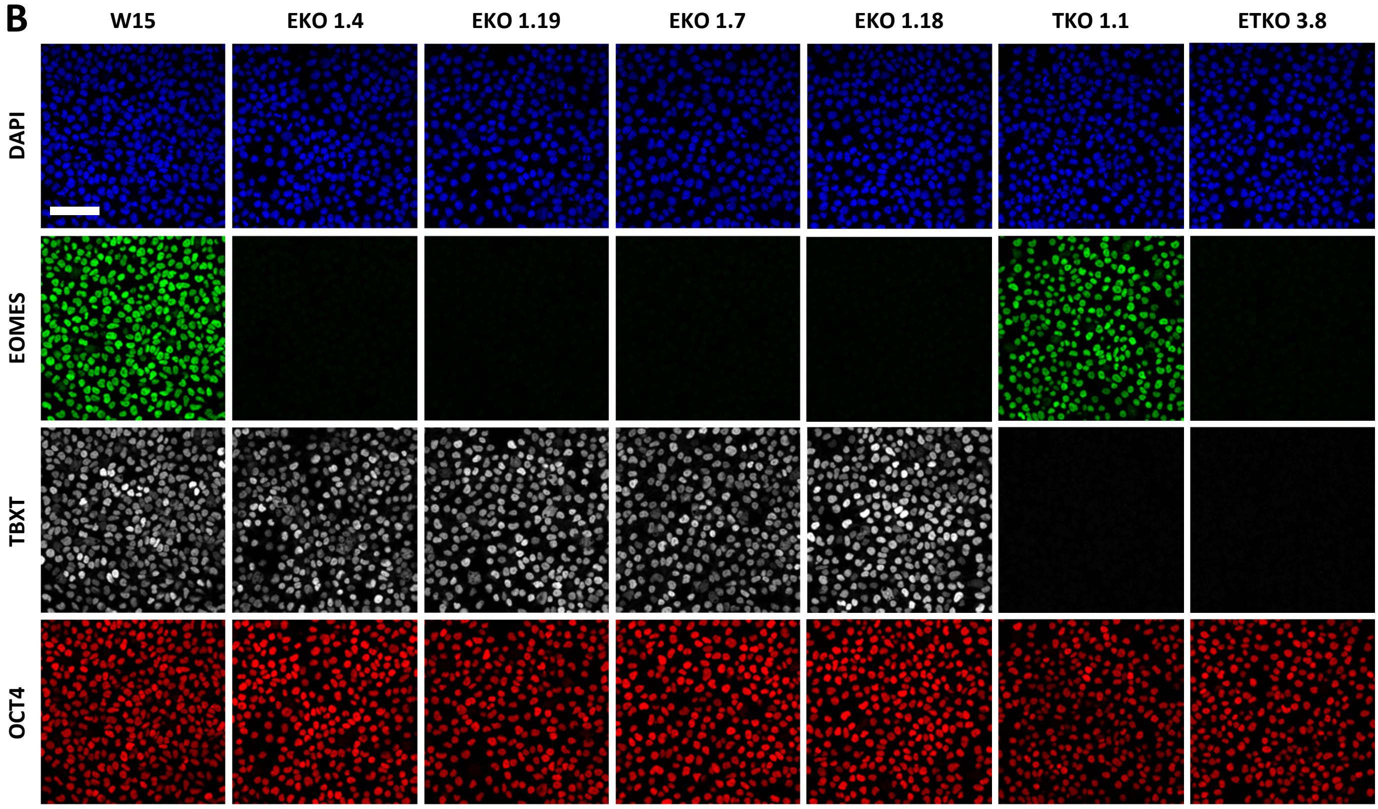

Figure S13. Characterization of EOMES, TBXT, and EOMES/TBXT knockout lines, Related to Figure 5
(A) Bright-field imaging for parental (W15), EOMES (EKO), TBXT (TKO) and EOMES/TBXT (ETKO) knockout clones in primed (E8), peri-gastrulation (4i and PreME), and resetting (tt2iGöXAV and HENSM) culture conditions. (B) Immunofluorescence of EOMES, TBXT, and OCT4 for parental (W15), EOMES (EKO), TBXT (TKO) and EOMES/TBXT (ETKO) knockout clones cultured in mesoderm differentiation conditions for one day. DAPI nuclear counterstain showed in blue. Scale bar, 100 um.

Figure S14 (supports figure 5)

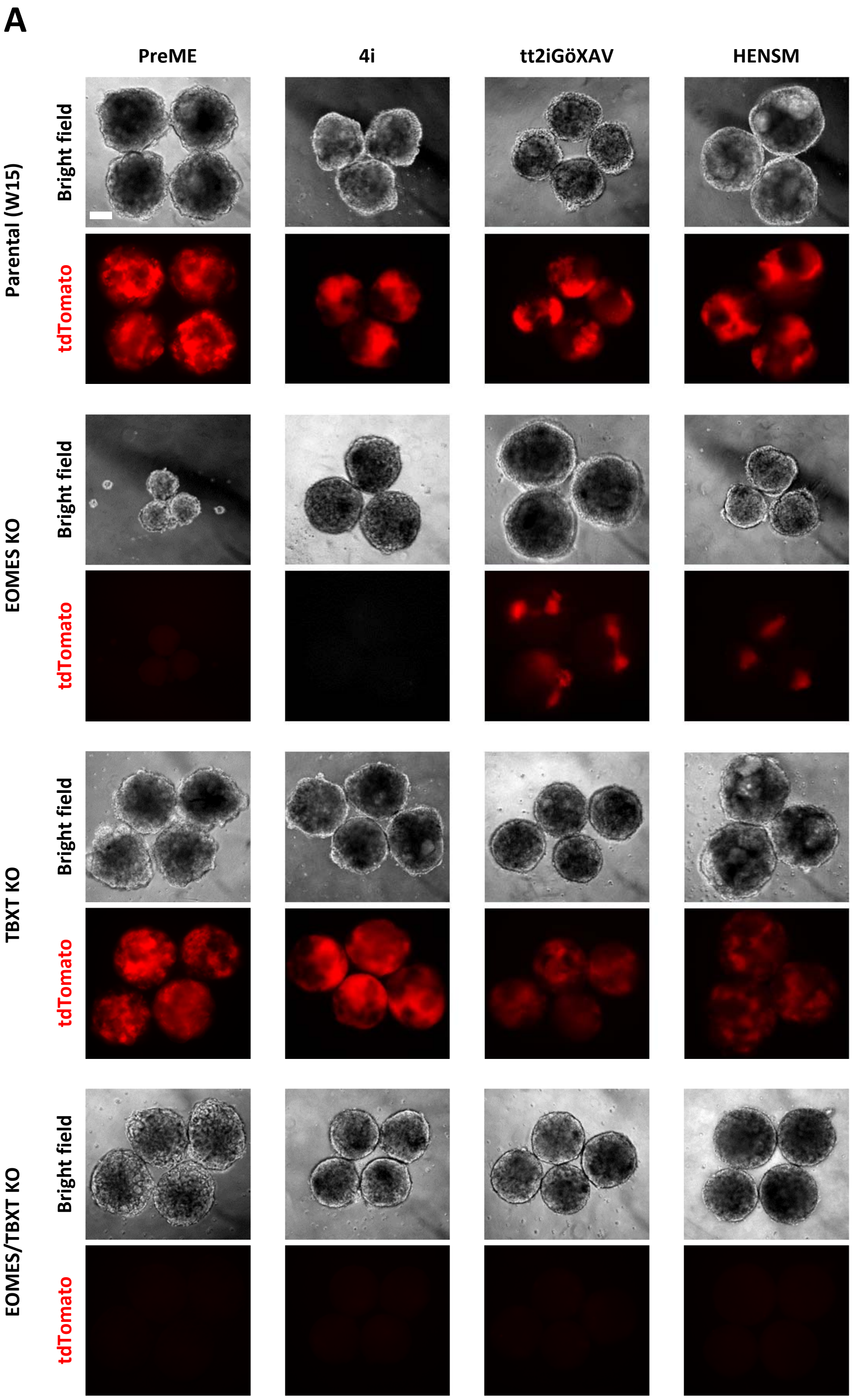

Figure S14. Characterization of hPGCLC competence from EOMES, TBXT, and EOMES/TBXT knockout lines (embryoid bodies), Related to Figure 5 (A) Day 4 embryoid bodies generated from parental (W15), EOMES, TBXT and EOMES/TBXT knockout (KO) NANOS3−tdTomato hESCs cultured in peri-gastrulation (4i and PreME) and resetting (tt2iGöXAV and HENSM) conditions . hPGCCs in the embryoid bodies expressed NANOS3−tdTomato. Scale bar: 200 um.

Figure S15 (supports figure 5)

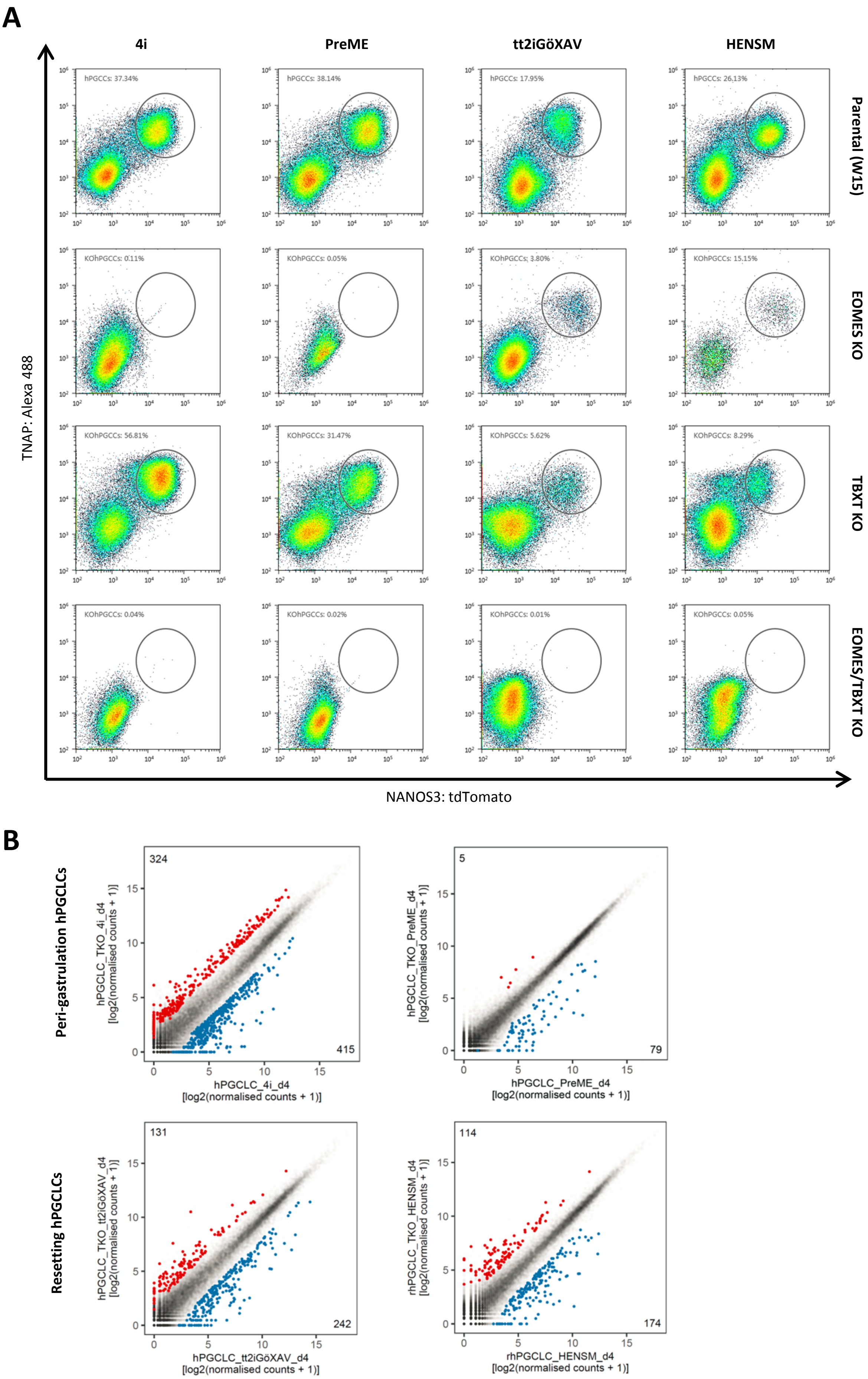

Figure S15. Characterization of hPGCLC competence from EOMES, TBXT, and EOMES/TBXT knockout lines (flow cytometry plots), Related to Figure 5 (A) Flow cytometry analysis plots showing the percentage of hPGCLCs co-expressing NANOS3−tdTomato and TNAP in day 4 embryoid bodies generated from peri-gastrulation (4i and PreME) and resetting (tt2iGöXAV and HENSM) hESCs in four different genetic backgrounds (parental (W15), EOMES, TBXT, and EOMES/TBXT knockout (KO) lines). (B) Scatter plots showing the number of DEGs ($log_2FC > 2$ and adjusted p-value < 0.05) between parental (W15) and TBXT knockout (TKO) hPGCLCs specified from peri-gastrulation (4i and PreME) and resetting (tt2iGöXAV and HENSM) precursors.

Figure S16 (supports figure 5)

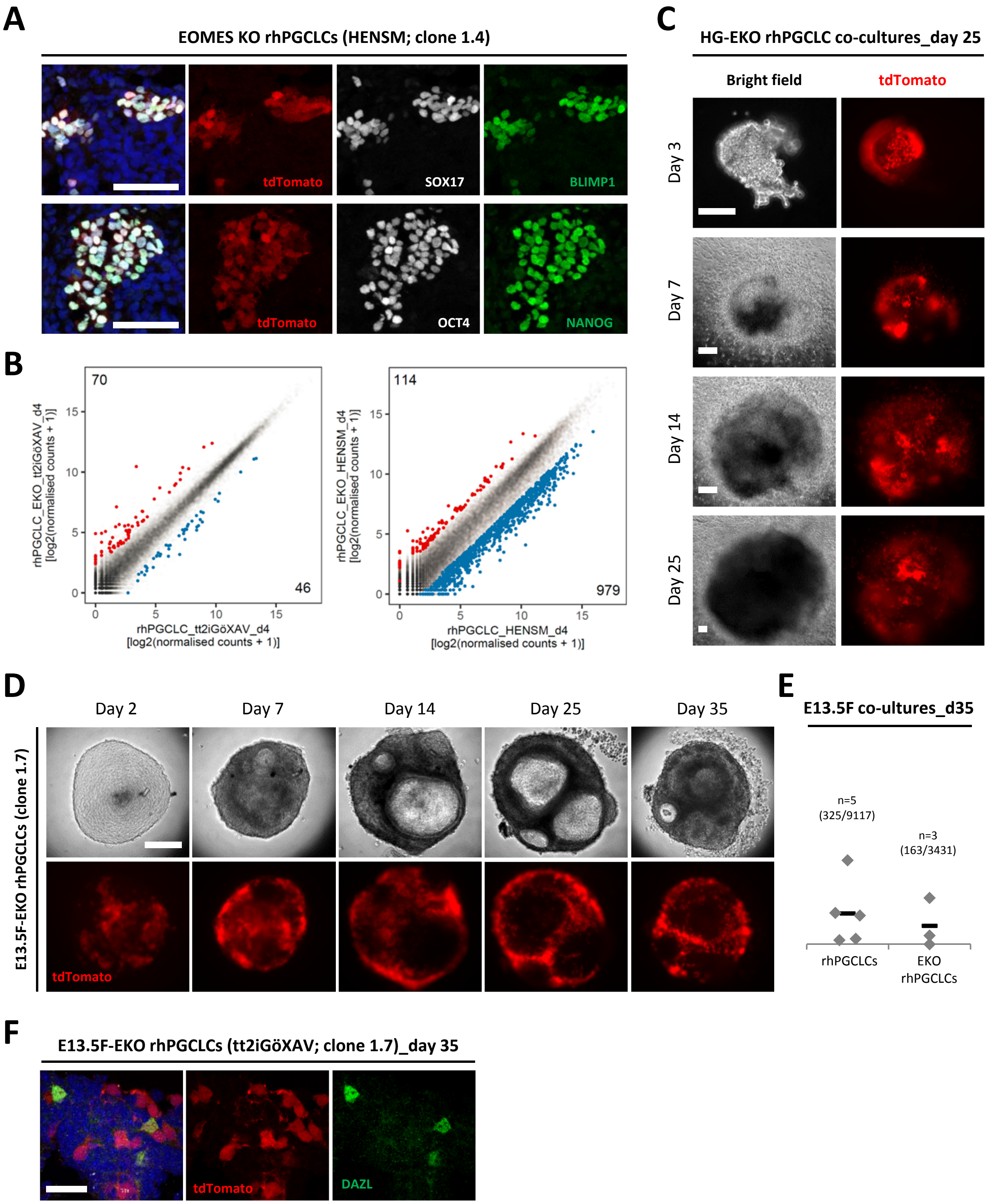

Figure S16. Progression of EOMES knockout resetting hPGCLCs supported by human hindgut organoids or mouse female gonadal somatic cells, Related to Figure 5 (A) Immunofluorescence of OCT4, SOX17, NANOG and BLIMP1 on sections from day 4 embryoid bodies containing rhPGCLCs expressing NANOS3−tdTomato specified from EOMES knockout (KO) resetting hESCs (HENSM; clone 1.4). DAPI nuclear counterstain showed in blue. Scale bar, 50 um. (B) Scatter plots showing the number of DEGs (log_2FC > 2 and adjusted p-value < 0.05) between parental (W15) and EOMES knockout (EKO) hPGCLCs specified from resetting (tt2iGöXAV and HENSM) precursors. (C) Co-culture of human hindgut organoid (HG) and EOMES knockout (EKO) NANOS3−tdTomato rhPGCLCs (tt2iGöXAV; clone 1.18) over 25 days. Scale bar, 200 um. (D) Co-culture of mouse E13.5 ovarian somatic cells (E13.5F) and EOMES knockout (EKO) NANOS3−tdTomato rhPGCLCs (tt2iGöXAV; clone 1.7) over 35 days. Scale bar, 200 um. (E) Percentage of DAZL positive tt2iGöXAV rhPGCLCs [wildtype and EOMES knockout (EKO)] out of NANOS3-tdTomato positive hPGCLCs in mouse E13.5 ovarian somatic cells (E13.5F) over 35 days. Horizontal bars represent the mean percentage for each condition. At least n=3 measurements were taken from independent experiments for each condition. Total number of cells counted (double DAZL and tdTomato positive/ tdTomato positive cells) per condition shown between brackets. (F) Immunofluorescence of DAZL on a section of day 35 mouse E13.5 ovarian somatic cell (E13.5F) co-cultures containing EOMES knockout (EKO) NANOS3−tdTomato rhPGCLCs (tt2iGöXAV; clone 1.7). DAPI nuclear counterstain showed in blue. Scale bar, 25 um.
